# Memory programming in CD8^+^ T-cell differentiation is intrinsic and is not determined by CD4 help

**DOI:** 10.1038/ncomms8994

**Published:** 2015-08-14

**Authors:** Juhyun Kim, Su Jeong Ryu, Keunhee Oh, Ji-Min Ju, Ji Yeong Jeon, Giri Nam, Dong-Sup Lee, Hang-Rae Kim, Joo Young Kim, Jun Chang, Thomas Sproule, Kyungho Choi, Derry Roopenian, Eun Young Choi

**Affiliations:** 1 Department of Biomedical Sciences, Seoul National University College of Medicine, Seoul 110-799, Korea; 2 Division of Life and Pharmaceutical Sciences, Ewha Womans University, Seoul 120-750, Korea; 3 The Jackson Laboratory, Bar Harbor, Maine ME04609, USA

## Abstract

CD8^+^ T cells activated without CD4^+^ T-cell help are impaired in memory expansion. To understand the underlying cellular mechanism, here we track the dynamics of helper-deficient CD8^+^ T-cell response to a minor histocompatibility antigen by phenotypic and *in vivo* imaging analyses. Helper-deficient CD8^+^ T cells show reduced burst expansion, rapid peripheral egress, delayed antigen clearance and continuous activation, and are eventually exhausted. Contrary to the general consensus that CD4 help encodes memory programmes in CD8^+^ T cells and helper-deficient CD8^+^ T cells are abortive, these cells can differentiate into effectors and memory precursors. Importantly, accelerating antigen clearance or simply increasing the burst effector size enables generation of memory cells by CD8^+^ T cells, regardless of CD4 help. These results suggest that the memory programme is CD8^+^ T-cell-intrinsic, and provide insight into the role of CD4 help in CD8^+^ T-cell responses.

## Abstract (web summary)

Persistent antigen stimulation can cause exhaustion and unresponsiveness of CD8 cells, impairing the immune response. Here the authors show that increasing the number of CD8 cells, decreasing the antigen load or providing CD4 help can overcome the exhaustion and establish a memory response.

## Graphical abstract

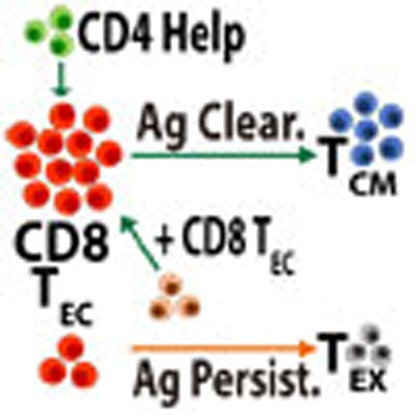

Stimulation of CD8^+^ T cells in the absence of CD4^+^ T-cell help is an important constraint on the quantity and quality of the CD8^+^ T-cell response, resulting in defects in memory expansion of activated CD8^+^ T cells^1^. The general consensus is that CD4 help delivered during CD8^+^ T-cell priming encodes a programme in the activated CD8^+^ T cells to generate memory cells^2^,^3^,^4^. CD4^+^ T cells provide paracrine cytokines and condition dendritic cells (DCs) to produce cytokines such as interleukin (IL)-12 and IL-15, express CD70 and increase antigen presentation, which enhance effector differentiation, proliferation and/or survival of the activated CD8^+^ T cells^5^,^6^,^7^,^8^,^9^,^10^,^11^. Nevertheless, what is the fundamental role of CD4^+^ T cells in preventing memory impairment of CD8^+^ T cells remains to be elucidated.

The strict requirement of CD4 help to drive CD8^+^ T-cell responses is most evident under non-inflammatory conditions modelled by immune responses to cellular antigens, such as minor histocompatibility (H) and tumour antigens. Antigen-specific CD8^+^ T cells primed under helper-deficient conditions were shown to be defective in clonal expansion and functional activation, and become non-responsive (tolerant) to antigen re-encounters^12^,^13^,^14^,^15^. However, the reliance on contrived approaches to create helper deficiency, such as CD4 depletion and the use of major histocompatibility complex (MHC) II- or CD4-deficient mice, and the paucity of antigen-specific CD8^+^ T cells expanded after helper-deficient activation limit extrapolating these results to physiological situations. Most of all, how tolerance is implemented in CD8^+^ T cells activated without CD4^+^ T-helper cells is not understood.

To address the helper-dependent nature of the CD8^+^ T-cell response under physiological conditions using natural cellular model antigens, we exploited a system in which the CD8^+^ T-cell response is induced against a single minor H epitope, H60. Minor H antigens are naturally processed peptides with a polymorphism at the epitope fragments presented by MHC^16^ and recognized as foreign epitopes after allogeneic transplantation. H60 is notably immunodominant, since a single H-2K^b^-presented H60 peptide (LTFNYRNL) elicits a CD8^+^ T-cell response dominating the responses to other minor H antigens, as seen in a C57BL/6 (B6) mice immunized with splenocytes from BALB.B mice that express the same MHC genes (H-2^b^-matched) with but different background genes (minor H antigen-mismatched) from those of B6 mice^17^. However, this immunodominance is CD4^+^ T-helper cell-dependent. Thus, the specific CD8^+^ T-cell response becomes subservient in the absence of concomitant activation of CD4^+^ T cells^18^. This critical feature provided the rationale for our use of H60 as a model antigen to investigate the effects of CD4^+^ T cells on the CD8^+^ T-cell response.

The B6.CH60 mouse strain has congenic *H60* region in a B6 background on chromosome 10. This region provides the H60-CD8 epitope to T cells in the B6 strain, which does not express H60 (ref. 19). The male Y chromosome of both strains contains the *Hy-Dby* locus, which provides the CD4 epitope (NAGFNSNRANSSRSS/H-2A^b^) to female B6 T cells^20^. Hence, transplanting spleen cells from male or female B6.CH60 mice to female B6 mice could generate a helped or helper-deficient H60-specific CD8^+^ T-cell response, respectively, in host female B6 mice^21^. Using this system, we have reported the requirement for CD40-CD40L-mediated CD4 help in the induction of primary and memory expansions of H60-specific CD8^+^ T cells^21^,^22^, and recruitment of diverse T-cell receptors (TCRs) to the specific CD8^+^ T-cell response^23^.

To understand the cellular mechanisms underlying the impaired memory in CD8^+^ T cells activated without CD4 help, we longitudinally characterized the response developed by helper-deficient CD8^+^ T cells using the H60 congenic mouse system. Here we provide evidence that the programme for central memory (T_cm_) generation is preserved intrinsically in CD8^+^ T cells.

## Results

### Tolerance of CD8^+^ T cells primed in the absence of CD4 help

**Figure 1:**
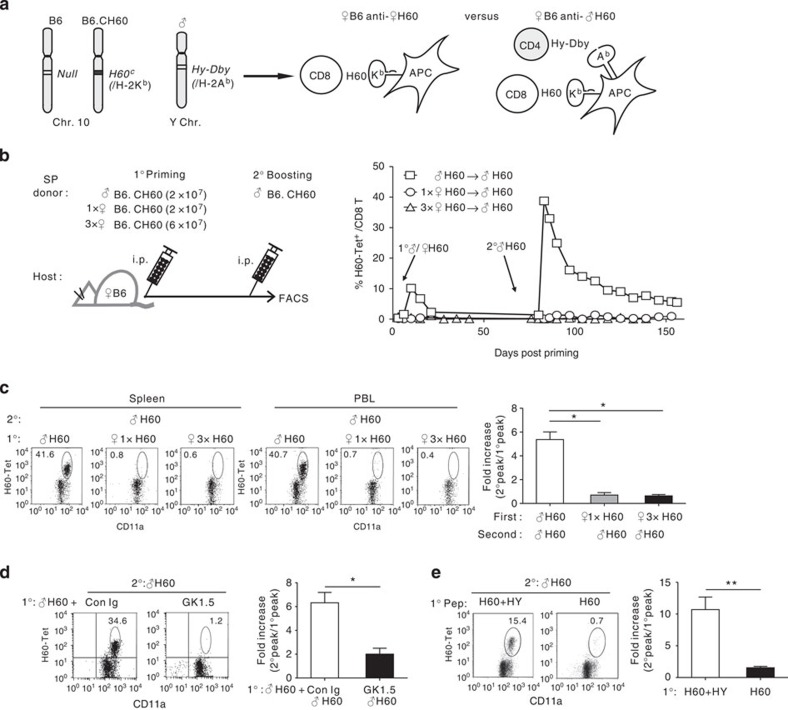
Impairment of memory expansion of H60-specific CD8^+^ T cells activated in the absence of CD4^+^ T-cell help. (**a**) Diagrams of experimental systems using B6.CH60 mice. The B6 mouse strain has null expression from the H60 locus, whereas the B6.CH60 strain expresses *H60*^*C*^ allele at the locus, which produces the H-2K^b^-restricted CD8 epitope to activate T cells of B6 mice. The *Hy-Dby* gene on the Y chromosome encodes the CD4 epitope presented by H-2A^b^. Priming of female B6 mice with female or male cells from B6.CH60 mice induces the helper-deficient or helped response for H60, respectively. (**b**) Female B6 mice were primed with total splenocytes of female B6.CH60 mice at a 1 × dose (2 × 10^7^) or a 3 × dose (6 × 10^7^), or with male (2 × 10^7^) B6.CH60 spleen cells, and then boosted with male B6.CH60 spleen cells (2 × 10^7^). Pooled PBLs from three immunized mice were analysed periodically using flow cytometry after staining with H60 tetramers, and anti-CD11a and anti-CD8 monoclonal antibodies (mAbs). The frequencies of H60-tetramer-binding cells in blood CD8^+^ T cells were then plotted. (**c**) Flow cytometric data of splenocytes and PBLs obtained on day 7 post boosting are shown after CD8^+^ gating, with percentages of the H60-tetramer-binding cells in CD8^+^ T cells denoted. The fold increase in the frequency of the secondary peak (day 7 post boosting) relative to that of the primary peak (day 10 post priming) is shown. (**d**) Female B6 mice treated with GK1.5 (for CD4 depletion) or control Ig were primed with male B6.CH60 cells. (**e**) Female B6 mice were primed with female syngeneic cells loaded with the H60 peptide alone or combined with the HY-Dby peptide. Then, these primed mice (**d**,**e**) were boosted with male B6.CH60 cells 40 days later. Representative data from flow cytometric analysis of PBLs from the boosted mice on day 7 post boosting are shown, with the plots of the fold increase in the frequency. All data (**b**–**e**) are representative of more than three independent experiments (*n*=3 per group per experiment). Data are presented as means±s.e.m. *P<0.05, **P<0.01 determined by Student's *t*-test.

Our previous study demonstrated that cell-fate decisions regarding CD8^+^ T-cell responsiveness to secondary challenge occur during the primary response^22^. Therefore, we examined whether H60-specific CD8^+^ T cells primed without CD4^+^ T-cell help would be predestined to become non-responsive to antigen re-encounter. Thus, female B6 mice primed previously with female B6.CH60 spleen cells (2 × 10^7^ cells; helper-deficient priming) were boosted with male B6.CH60 splenocytes and traced longitudinally to detect H60-specific CD8^+^ T cells in blood via H60-tetramer staining (Fig. 1a,b). H60-tetramer-binding CD8^+^ T cells were scarce in the blood and spleen (<1.2% of CD8^+^ T cells) of helper-deficient primed mice even after boosting, whereas mice primed with an equal number of male B6.CH60 splenocytes (2 × 10^7^ cells; helped priming) showed a five- to sevenfold increase in peak frequencies (∼40%) compared with the primary peaks on boosting (Fig. 1b,c). Such limited responsiveness of CD8^+^ T cells in helper-deficient primed mice was recapitulated when the priming and boosting numbers of B6.CH60 splenocytes were reduced (5 × 10^6^ and 3 × 10^5^; Supplementary Fig. 1).

A lack of secondary expansion was also observed when helper-deficient conditions were generated by antibody (GK1.5)-mediated pre-depletion of CD4^+^ T cells in the hosts or by priming hosts with female cells loaded with H60 peptide alone without co-loading the HY-Dby CD4 epitope–peptide (Fig. 1d,e). These results confirmed that the memory defect was based on recognition of the H60 peptide/MHC complex by specific CD8^+^ T cells in the absence of concomitantly activated CD4^+^ T cells.

As the magnitude of the CD8^+^ T-cell response positively correlated with epitope levels^24^,^25^, we examined the effect of threefold increase (3 × ) in the priming dose under helper-deficient conditions. However, elevating the antigen dose did not compensate for the CD4 help deficiency, but tended to further reduce responsiveness of the specific CD8^+^ T cells to H60 re-encounter (Fig. 1b,c). Thereafter, we primed mice under 3 × helper-deficient conditions to ensure generation of memory impairment and boosted 40 days later, which are referred to as tolerizing and boosting schedules, respectively. Thus, priming the female H60-specific CD8^+^ T cells without help from male-specific CD4^+^ T cells provided an appropriate model system for studying the mechanism underlying establishment of memory impairment (or tolerance) in helper-deficient activated CD8^+^ T cells.

### Functional impairment of helper-deficient CD8^+^ T cells

**Figure 2:**
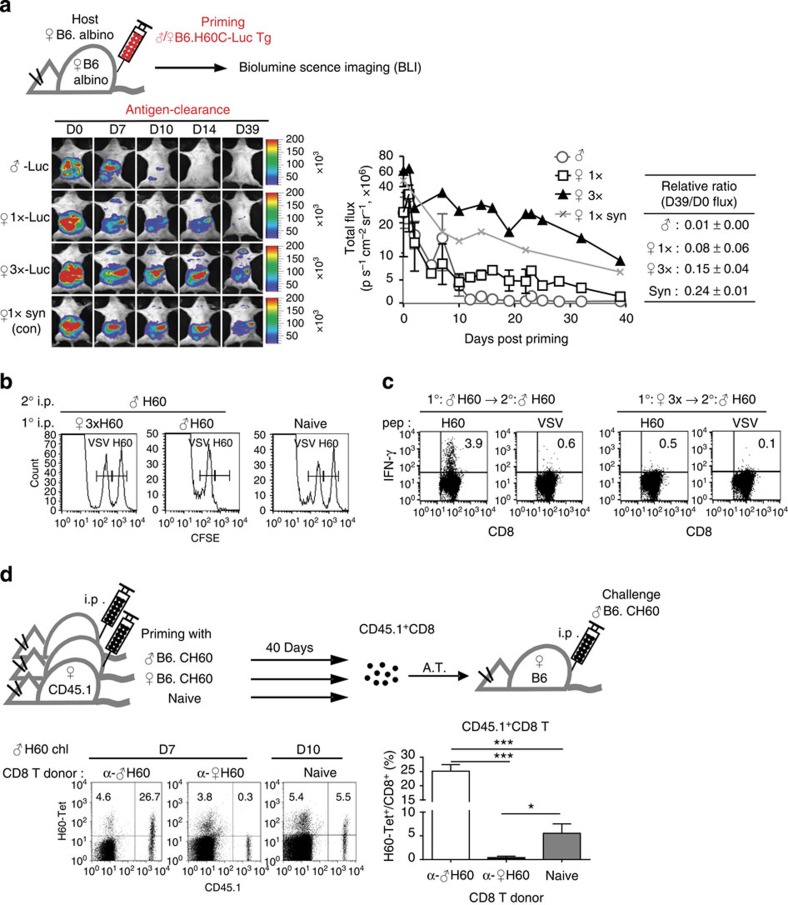
Ineffective antigen clearance and CD8^+^ T-cell-intrinsic memory impairment under helper-deficient conditions. (**a**) *In vivo* imaging of H60 allografts (B6.CH60-LucTg cells). Female B6-Albino hosts were i.p. injected with female or male B6.CH60-LucTg cells, and bioluminescence images were captured periodically. Female B6.CH60-Albino hosts were primed with B6.CH60-LucTg cells as syngeneic controls. The whole body was designated as the region of interest (ROI), and the photon flux outcomes from the ROIs along the response progress were plotted. The mean relative ratios of day 39: day 0 photon flux values are presented in the table. (**b**) *In vivo* cytotoxicity assay. Mixtures (1:1) of the CFSE-labelled and peptide-loaded target cells (2.5 μM CFSE-labelled and H60-loaded versus 0.42 μM CFSE-labelled and VSV-loaded) were i.v. injected into each group of mice and analysed 72 h later by flow cytometry of the PBLs for detection of the CFSE-labelled cells. (**c**) Specific IFN-γ production was assessed in splenocytes harvested from tolerized-and-boosted or helped-and-boosted hosts on day 7 post boosting. (**d**) CD45.1^+^ female B6 mice were primed with female or male B6.CH60 splenocytes 40 days previously. Then, CD8^+^ T cells (3 × 10^6^) purified from these differently primed CD45.1^+^ mice or those purified from unprimed naive CD45.1^+^ mice were adoptively transferred (A.T.) to naive female CD45.2^+^ B6 mice. Then, the naive adoptive hosts were challenged with male B6.CH60 splenocytes. PBLs were longitudinally analysed after gating on CD8^+^ cells. Representative flow cytometric data obtained on day 7 or 10 post challenge are presented. Proportions of H60-Tet^+^ cells in CD45.1^+^ and CD45.1^−^ CD8^+^ cells are denoted in the upper quadrants and the percentage values in the CD45.1^+^CD8^+^ cells are plotted. Representative data from more than three independent experiments (*n*=3 per group per experiment) are shown (**a–d**). Data are presented as means±s.e.m. *P<0.05, ***P<0.001 determined by Student's *t*-test.

Next, we tested whether the helper-deficient CD8^+^ T cells were functionally abortive. To trace rejection of H60-primary allografts (B6.CH60 splenocytes; antigen clearance) *in vivo*, longitudinal bioluminescence imaging (BLI) analysis was performed, using luciferase transgenic mice^26^ backcrossed to B6.CH60 mice (B6.CH60-LucTg) as spleen cell donors. Then, the decline in signals emitted by the injected B6.CH60-LucTg cells was monitored as an indication of the antigen clearance. BLI data showed that the decrease in signal in 1 × helper-deficient hosts was slow, while it rapidly dropped after day 7 and eventually disappeared in the helped hosts; this occurred more rapidly than in hosts injected with syngeneic control cells, which maintained the signal over a long period (Fig. 2a). The delay in antigen clearance under helper-deficient conditions was further verified using flow cytometry, in which primed B6.H60 splenocytes were consistently detected in the blood of the helper-deficient hosts long after priming even with low antigen doses (Supplementary Fig. 1). In the 3 × helper-deficient hosts, signal decline was maintained further; very high signals were detected on day 39 post priming, reflecting the heavy antigen load. These data illustrated the ineffectiveness in antigen clearance by helper-deficient CD8^+^ T cells.

The lack of functional activity of H60-specific CD8^+^ T-cell in rechallenge phase of the tolerized mice was confirmed by *in vivo* cytotoxicity and *ex vivo* interferon (IFN)-γ-secretion assays (Fig. 2b,c).

Since the antigen (H60 allografts) persisted in the helper-deficient hosts, we examined whether the tolerance of the helper-deficient CD8^+^ T cells to H60 re-encounter was CD8^+^ T-cell-intrinsic or derived from the helper-deficient host environment. CD8^+^ T cells purified from the tolerized CD45.1^+^ mice were adoptively transferred into new naive CD45.2^+^ mice (Fig. 2d). After challenging the adoptive hosts with male B6.CH60 splenocytes, peripheral blood leukocyte (PBL) analysis showed that the CD45.1^+^ CD8^+^ T cells originating from helper-deficient hosts did not respond to the H60 re-encounter even in the new naive environment, while those that originated from helped or naive mice expanded properly in response to H60 stimulation (Fig. 2d). Thus, the memory impairment of helper-deficient CD8^+^ T cells was CD8^+^ T-cell-intrinsic.

### Effector memory status of helper-deficient CD8^+^ T cells

**Figure 3:**
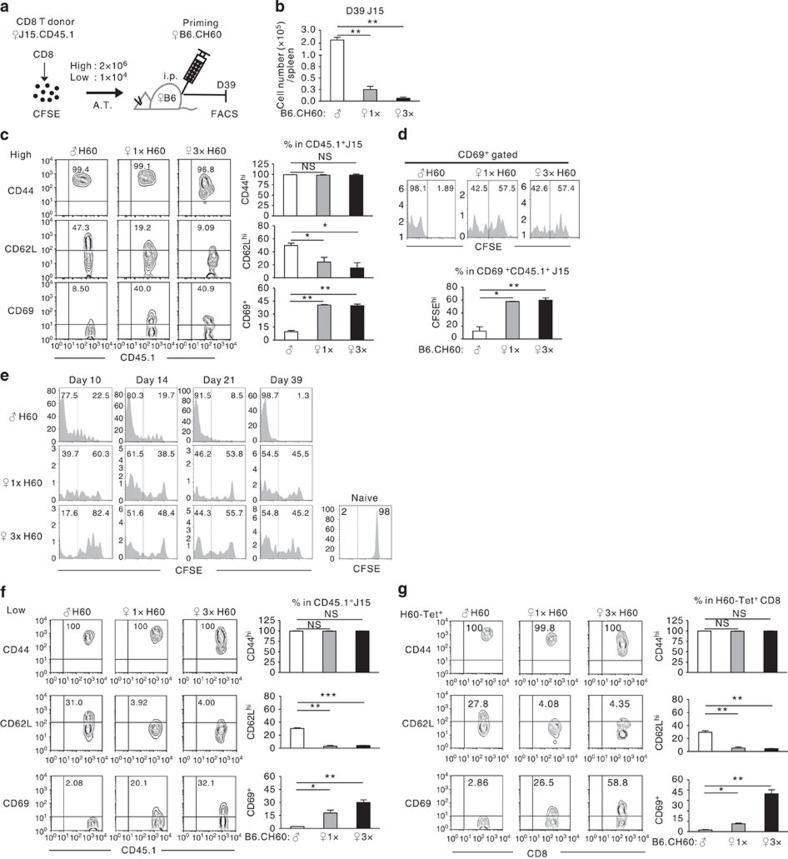
Memory phenotypes of H60-specific CD8^+^ T cells under different conditions. (**a**) Schematic presentation of the experimental procedure for analysis of the helped and helper-deficient H60-specific CD8^+^ T cells. CFSE-labelled CD45.1^+^ J15 CD8^+^ T cells were adoptively transferred (A.T.) into female B6 mice at a high (2 × 10^6^) or low (1 × 10^4^) number. Then, the adoptive hosts were primed under helped or helper-deficient conditions (1 × and 3 × helper-deficient conditions). Splenic J15 CD8^+^ T cells harvested on day 39 post priming were analysed. (**b**) The numbers and (**c**) representative flow cytometric data with plots of the proportions of different populations in J15 CD8^+^ T cells harvested from spleens of adoptive hosts with higher number transfer (high) are shown. (**d**) CFSE profiles after gating for CD69^+^cells, and the proportion of CFSE^hi^ populations in the CD69^+^ CD45.1^+^ J15 CD8^+^ T cells are shown. (**e**) CFSE-dilution peaks of J15 CD8^+^ T cells harvested at the indicated time points. The CFSE profile of naive J15 CD8^+^ T cells on day 21 after PBS injection into adoptive hosts is shown as the naive control. (**f**,**g**) Phenotypic analyses of (**f**) splenic J15 CD8^+^ T cells after low-number transfer (1 × 10^4^; low) and (**g**) polyclonal H60-tetramer-binding CD8^+^ T cells magnetic-activated cell sorting-purified from spleens of primed B6 hosts (without J15 transfer; H60-Tet^+^) were performed on day 39 post priming. Mice in each experimental group (**b**–**g**) were analysed individually (*n*=2 per group). Flow cytometric results were analysed after gating for CD45.1^+^CD8^+^ cells (**b**–**f**) or CD8^+^ cells (**g**). All data (**b**–**g**) are representative of more than three independent experiments. The values from all experiments were incorporated into plots (**b**–**g**). Data are presented as means±s.e.m. Not Significant (NS) *P*>0.05. *P<0.05, **P<0.01, ***P<0.001 determined by Student's *t*-test.

To characterize the CD8^+^ T cells in which tolerance was implemented, phenotypes of the helper-deficient and helped CD8^+^ T cells were compared on day 39 post priming, 1 day before boosting. To circumvent the limitation in numbers of polyclonal H60-specific CD8^+^ T cells obtainable at the memory phase of helper-deficient response, we generated a TCR transgenic mouse (Tg) line named J15 in which T cells express TCRs originating from a previously characterized H60-specific CTL clone^27^ (Supplementary Fig. 2). The CD8^+^ T cells from J15 mice on the CD45.1^+^ background (J15 CD8^+^ T cells) were labelled with carboxyl fluorescein diacetate succinimidyl ester (CFSE) and adoptively transferred into CD45.2^+^ B6 mice (Fig. 3a). Although high numbers of J15 CD8^+^ T cells were transferred (2 × 10^6^) to facilitate analysis on day 39, the numbers of splenic J15 CD8^+^ T cells harvested from both helped and helper-deficient hosts remained low (Fig. 3b). While ∼50% of the helped cells were CD62L^hi^CD44^hi^ T_cm_, most helper-deficient T cells were CD62L^low^CD44^hi^ effector (T_eff_) or effector memory (T_em_) cells, consistent with the phenotype of CD8^+^ T cells observed in the memory phase after infection of *Cd4*^−/−^ mice with lymphocytic choriomeningitis virus^28^. Notably, ∼40% of the helper-deficient T cells remained CD69-positive (Fig. 3c). About 50% of the CD69^+^ cells were CFSE^hi^ (Fig. 3d), indicating new activation of CD8^+^ T cells from naive, as well as antigen-experienced pools, by the remaining antigen in the memory phase. Consistently, we observed CFSE^hi^ populations in the helper-deficient splenic J15 CD8^+^ T cells on days 10, 14 and 21 post priming (Fig. 3e).

The precursor frequency of H60-specific CD8^+^ T cells was estimated to be 1/20,000–1/10,000 (ref. 18). To exclude the effect of J15 cells present at high frequency in the naive T-cell pool, we reduced the number of transferred cells to physiological levels (0.1–1 × 10^4^)^29^. We observed the same T_em_/T_eff_ phenotype and the presence of CD69^+^ cells with the helper-deficient J15 CD8^+^ T cells transferred in low numbers, 10^4^ and 10^3^ (Fig. 3f and Supplementary Fig. 3). Finally, analysis of cells purified from splenocytes of primed B6 mice (without the J15 T-cell transfer) also demonstrated that the polyclonal H60-tetramer-binding CD8^+^ T cells in the helper-deficient hosts were of identical phenotype (Fig. 3g). Therefore, the H60-specific CD8^+^ T cells were continuously recruited to the helper-deficient responses and were subsequently adopting a T_em_/T_eff_ phenotype.

### Exhaustion of helper-deficient CD8^+^ T cells

**Figure 4:**
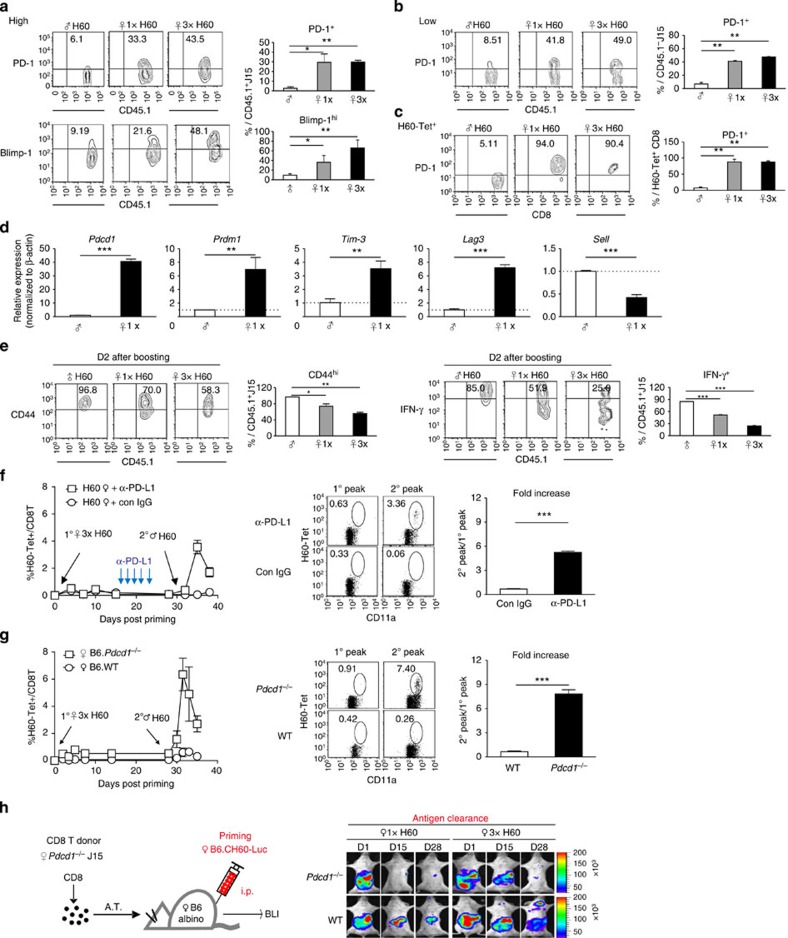
Exhaustion of H60-specific CD8^+^ T cells under helper-deficient conditions. (**a**) Flow cytometric analysis of J15 CD8^+^ T cells (transferred in high numbers) for expression of PD-1 and Blimp-1 on day 39 post priming (*n*=2 per group per experiment). (**b**,**c**) Flow cytometric analyses of (**b**) J15 CD8^+^ T cells transferred in low numbers and (**c**) polyclonal H60-tetramer-binding CD8^+^ T cells on day 39 post priming. Percentages of the positive population are plotted (*n*=2 per group). (**d**) Real-time quantitative RT–PCR analysis to determine relative expression levels of *Pdcd1* (PD-1), *Prdm1* (Blimp-1), *Tim-3* (Tim-3), *Lag3* (Lag3) and *Sell* (CD62L). RNA was isolated from splenic J15 CD8^+^ T cells in helped or helper-deficient adoptive hosts transferred in high numbers on day 39 post priming. Delta–delta *C*_t_ values were normalized to those obtained from amplification of *β-actin* and were expressed as fold changes compared with helped J15 gene profiles. Data represent the means±s.e.m. of two independent experiments (*n*=5 per group). (**e**) Expressions of CD44 and IFN-γ by J15 CD8^+^ T cells on day 2 post boosting of the adoptive hosts transferred in high numbers. Percentages of positive cells in CD45.1^+^J15 CD8^+^ T cells are plotted (*n*=2 per group). (**f**) Helper-deficient primed mice were treated with anti-PD-L1 mAb or control Rat IgG2b once every 3 days from day 14 post priming (five times). The primed-and-antibody-treated mice were boosted on day 40 post priming (*n*=3 per group). (**g**) Female PD-1-deficient or WT B6 mice were challenged according to the tolerizing and boosting schedules (*n*=3 per group). (**f**,**g**) Longitudinal flow cytometric analysis of PBLs was performed and flow cytometric data obtained on day 10 post priming (1^o^ peak) and day 7 post boosting (2^o^ peak) are presented with plots of fold change in frequencies of H60-tetramer-binding CD8^+^ T cells. (**h**) BLI analysis of antigen clearance. B6-albino hosts adoptively transferred (A.T.) with *Pdcd1*^−/−^ or WT J15 CD8^+^ T cells were monitored periodically after 1 × or 3 × helper-deficient priming with B6.CH60-LucTg cells (*n*=2 per group). Data shown represent two (**b–f**) or at least three (**a**,**g**,**h**) independent experiments. Data are presented as means±s.e.m. *P<0.05, **P<0.01, ***P<0.001 determined by Student's *t*-test.

Antigen persistence in the helper-deficient hosts (Fig. 2a) and the above T_em_/T_eff_ phenotype in helper-deficient T cells reminded us of CD8^+^ T-cell exhaustion in chronically infected hosts^30^. Thus, we assessed the expression of PD-1 surface marker and the Blimp-1 transcription factor associated with CD8^+^ T-cell exhaustion^31^. The proportions of the PD-1^+^ and Blimp-1^hi^ populations in J15 CD8^+^ T cells on day 39 were higher in helper-deficient hosts than in helped hosts (Fig. 4a). The high proportions of PD-1^+^ cells in the helper-deficient T cells were verified using J15 CD8^+^ T cells transferred in low numbers and polyclonal H60-tetramer-binding CD8^+^ T cells (Fig. 4b,c and Supplementary Fig. 3). Quantitative reverse transcription–polymerase chain reaction analysis confirmed higher expression of genes (*Pdcd1, Prdm1, Tim-3* and *Lag3*) relevant to exhaustion^32^ by helper-deficient CD8^+^ T cells, compared with helped cells (Fig. 4d). Furthermore, helper-deficient J15 CD8^+^ T cells downregulated IFN-γ and CD44 after boosting (Fig. 4e), another characteristic of exhausted CD8^+^ T cells^33^.

As prevention of PD-1-signalling restores the proliferative and functional activity of exhausted CD8^+^ T cells^34^, we examined whether memory expansion could be restored by administering anti-PD-L1 antibody during the helper-deficient response. PBL analysis demonstrated that the frequencies of H60-tetramer-binding CD8^+^ T cells increased significantly after boosting the anti-PD-L1-treated group of helper-deficient hosts (Fig. 4f). Moreover, memory expansion of the H60-specific CD8^+^ T cells was restored in the helper-deficient primed PD-1-deficient (*Pdcd1*^−/−^) hosts (Fig. 4g). BLI analysis of the primed H60 allografts (B6.CH60-LucTg cells) showed that antigen clearance was accelerated in helper-deficient hosts adoptively transferred with *Pdcd1*^−/−^ J15 CD8^+^ T cells (Fig. 4h). These results indicated that tolerance of helper-deficient CD8^+^ T cells to the H60 re-encounters corresponded to the CD8^+^ T-cell exhaustion in association with long-term antigen persistence.

### Kinetics of H60-specific CD8^+^ T-cell response

**Figure 5:**
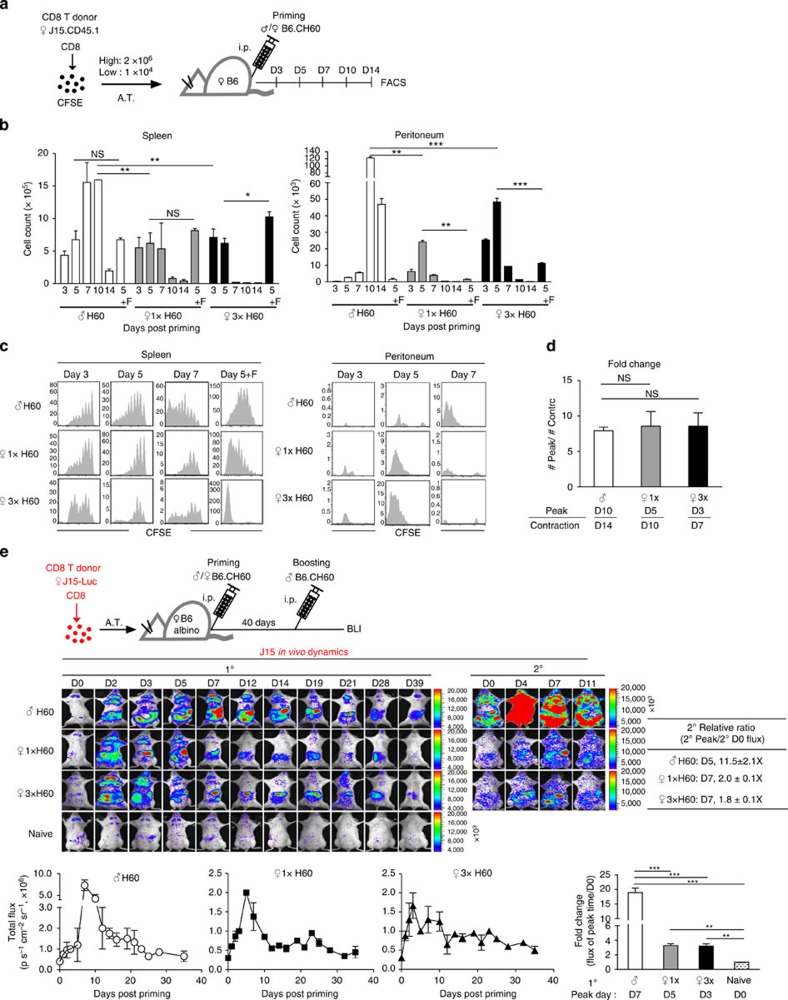
Dynamics of H60-specific CD8^+^ T cells under different conditions. (**a**) J15 CD8^+^ T cells were harvested from the spleen and peritoneum of adoptive hosts at the indicated times after exposure to different priming conditions, (**b**) quantified and (**c**) analysed by flow cytometry to compare CFSE-dilution profiles. Numbers and CFSE profiles of J15 CD8^+^ T cells from FTY-720-treated mice were obtained on day 5 post priming and were incorporated in plots designated as day 5+F in (**b**,**c**). (**d**) Contraction degrees (peak numbers/contraction numbers) of splenic J15 CD8^+^ T cells obtained from adoptive hosts are plotted. Data shown were obtained from adoptive hosts transferred in high numbers (**b–d**). (**e**) Dynamics of J15-LucTg CD8^+^ T cells in B6-albino-adoptive hosts (transferred with 1 × 10^5^ cells) were monitored periodically after priming under different conditions through secondary expansion. Those in B6-albino-adoptive hosts without priming are also shown as control (naive). Photon flux values as the primary responses progressed were plotted after designating the whole body as the ROI. Fold changes in peak values in 1^o^ peak relative to the values of 1^o^ day 0 (before priming) are shown. The mean relative ratios of 2^o^ peak: 2^o^ day 0 (before boosting) photon flux values are presented in the table. Data shown represent at least three (*n*=2 per group; **b–d**) or two (*n*=3 per group; **e**) independent experiments. A.T., Adoptive Transfer. Data are presented as means±s.e.m. Not Significant (NS) *P*>0.05, *P<0.05, **P<0.01, ***P<0.001 determined by Student's *t*-test.

As helper-deficient tolerization was implemented by helper-deficient priming, we reasoned that the core effects of CD4^+^ T-helper cells to prevent the induction of tolerance stemmed from the priming phase. Then, we analysed the characteristics of T-cell expansion, migration and differentiation after helper-deficient priming. We quantified CFSE-labelled CD45.1^+^J15 CD8^+^ T cells in the spleen, lymph nodes (LNs) and peritoneum of CD45.2^+^ adoptive hosts under different conditions, and compared the CFSE-dilution profiles and phenotypic characteristics at various time points post priming (Fig. 5a).

The numbers of J15 CD8^+^ T cells in the spleen peaked earlier in helper-deficient hosts during the expansion phase (days 5 and 3 under 1 × and 3 × conditions, respectively) than in the helped hosts (days 7–10), albeit at two- to threefold lower levels (Fig. 5b). The CFSE profiles of helper-deficient T cells in the spleen showed a relative absence of cells that had divided more than five times, particularly on days 5 and 7 post priming, suggesting that such cells would have either egressed or died, whereas the helped cells in the spleen exhibited profiles consistent with massive cell proliferation and accumulation (Fig. 5c). Similar dynamics were observed with J15 CD8^+^ T cells in the LNs (data not shown). However, analysis of J15 CD8^+^ T cells in the peritoneum, where the majority of B6.CH60 cells reside following the initial intraperitoneal (i.p.) injection, revealed that peritoneal J15 CD8^+^ T cells in helper-deficient hosts had undergone more than five cell divisions by day 5 post priming (Fig. 5c). Their numbers peaked earlier (day 5), with five- or threefold lower counts (under 1 × or 3 × conditions, respectively) than those of helped cells (day 10). In addition, when helper-deficient hosts were treated with FTY-720 to block cell egress from the lymphoid organs, CFSE^low^ J15 CD8^+^ T cells accumulated in the spleens on day 5 post priming (day 5+F; Fig. 5b,c), with few cells detected in the peritoneum (data not shown). This verified that helper-deficient J15 CD8^+^ T cells egressed from the spleen to the periphery after more than four or five divisions, rather than undergoing further proliferation within the spleen. After small burst of expansion, helper-deficient T cells contracted. The degrees of contraction (peak/contraction numbers) were in ordinary ranges, similar to those of helped cells (Fig. 5d). Only the time points of peak and contraction, and corresponding numbers differed, according to the kinetics of each response.

Primary expansion of luciferase-expressing J15 (J15-LucTg) CD8^+^ T cells in the lymphoid organs and peritoneum, although in small degrees, and impaired memory expansion in helper-deficient hosts were further visualized by longitudinal BLI analysis of helper-deficient primed hosts into which J15-LucTg CD8^+^ T cells were adoptively transferred (Fig. 5e).

**Figure 6:**
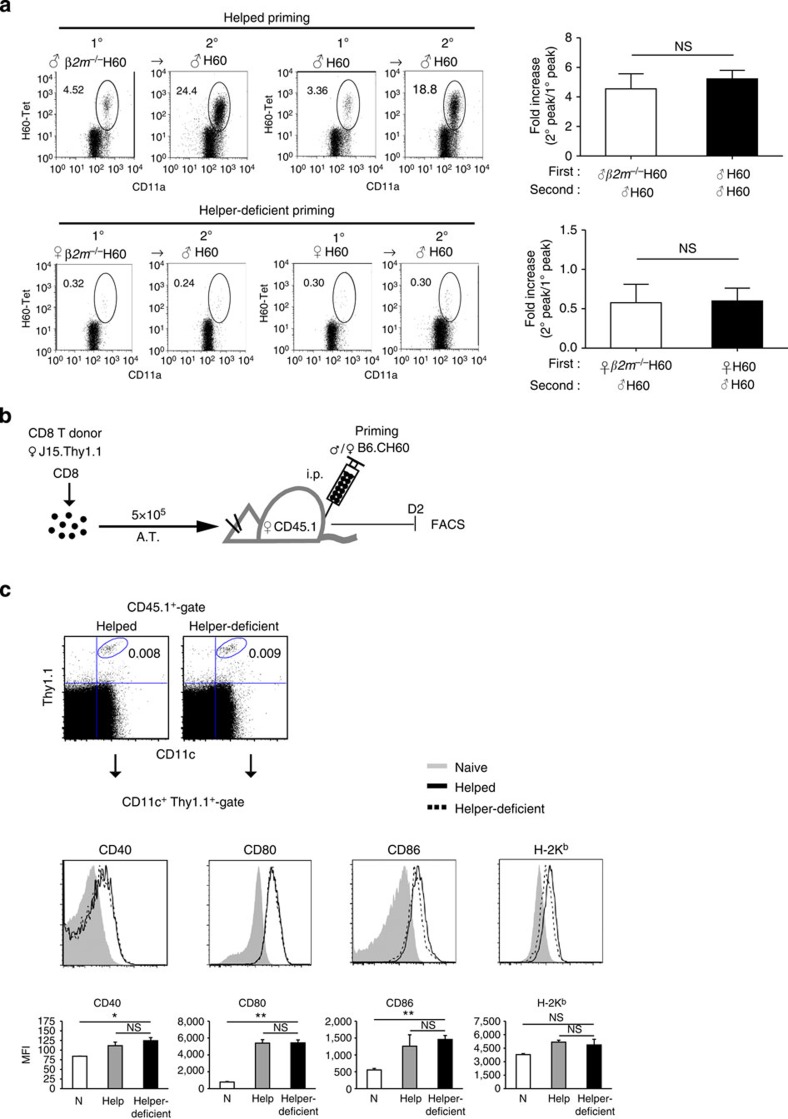
Host APCs participating in helper-deficient CD8^+^ T-cell activation. (**a**) Female B6 mice were primed with male or female *β2m*^−/−^B6.CH60 splenocytes and boosted with male B6.CH60 cells 40 days later. PBL analyses with H60-tetramer staining were performed longitudinally. The frequencies of the peaks during primary (1^o^; day 10 post priming) and secondary (2^o^; day 7 post boosting) responses were compared with those from mice primed and boosted with WT B6.CH60. The fold increases in peak frequencies between the primary and secondary responses were plotted. (**b**) Schematic presentation of the experimental procedure for analysis of the CD11c^+^ cells participating in helped and helper-deficient activation of H60-specific CD8^+^ T cells. Female CD45.1^+^ B6 mice were adoptively transferred with Thy1.1^+^ J15 CD8^+^ T cells (5 × 10^5^) and primed with male or female B6. CH60 splenocytes. Splenic DCs from primed hosts were analysed for expression of co-stimulatory molecules by flow cytometry on day 2 post priming. (**c**) CD45.1^+^CD11c^+^Thy1.1^+^ cells (host CD45.1^+^ CD11c^+^DCs in conjugates with Thy1.1^+^ J15 CD8^+^ T cells) were analysed for expression of CD40, CD80, CD86 and H-2K^b^. The levels under helped (black bold line) and helper-deficient (dashed lines) conditions were compared with reference to the levels on naive DCs (grey shaded). Average mean fluorescence intensity (MFI) values are plotted. Data are representative of three (**a**) or two (**c**) independent experiments (*n*=3 per group per experiment). A.T., Adoptive Transfer. Data are presented as means±s.e.m. Not Significant (NS) *P*>0.05, *P<0.05, **P<0.01 determined by Student's *t*-test.

The finding that helper-deficient CD8^+^ T cells could proliferate and migrate to the periphery, albeit to a small degree, raised the question whether the antigen-presenting cells (APCs) could deliver sufficient TCR and co-stimulatory signals to CD8^+^ T cells under helper-deficient conditions. Tolerization of H60-specific CD8^+^ T cells was mediated by host APCs rather than by the cells present in the immunizing donor B6.CH60 splenocytes, because the tolerance was still induced by immunization with female *β2m*^−/−^B6.CH60 splenocytes, which cannot directly present the H60 antigen (Fig. 6a). Host CD45.1^+^CD11c^+^ DCs participating in CD8^+^ T-cell activation during the helper-deficient priming phase were found to upregulate co-stimulatory molecules, such as CD40, CD80 and CD86, as well as the MHC-class I molecule (H-2K^b^) to the levels comparable to those under helped conditions (Fig. 6b,c). Thus, APCs under helper-deficient conditions were matured enough to stimulate the CD8^+^ T cells.

In summary, studies of CD8^+^ T-cell dynamics indicate that helper-deficient J15 CD8^+^ T cells were characterized by (i) fast kinetics, (ii) small burst expansion, (iii) intact capacity to migrate to peripheral antigen sites, being activated by adequately matured APCs and (iv) normal degrees of contraction. A threefold elevation in antigen load expedited the response progression further.

### Intact CD8^+^ effector differentiation without CD4 help

**Figure 7:**
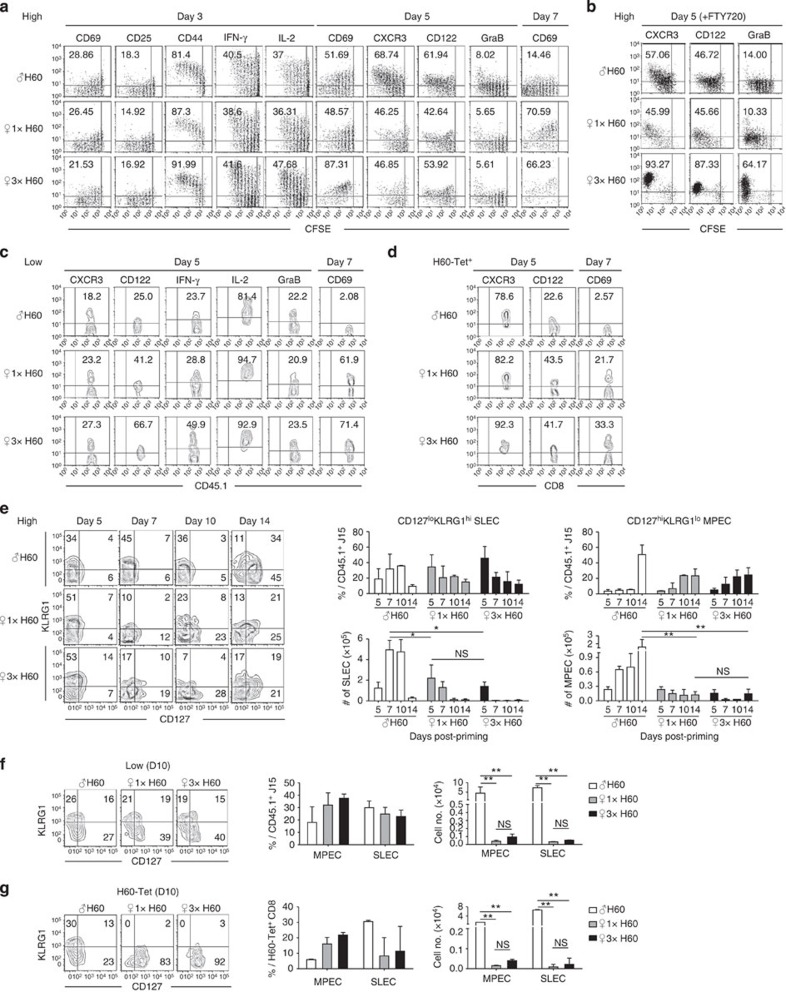
Effector differentiation between helper-deficient cells and helped cells. (**a**–**c**) Phenotypic profiles of CD45.1^+^ J15 CD8^+^ T cells in the spleens of adoptive hosts transferred in high numbers (**a**), treated with FTY-720 (**b**) or transferred in low numbers (**c**). (**d**) Phenotypic profiles of polyclonal H60-tetramer-binding CD8^+^ T cells. (**e–g**) Representative flow cytometry data and plots of the proportion and numbers of short-lived effector cell (SLEC) and memory precursor effector cell (MPEC) populations (**e**,**f**) in J15 CD8^+^ T cells from adoptive hosts transferred with high (**e**) or low (**f**) numbers of cells, or (**g**) in polyclonal H60-tetramer-binding CD8^+^ T cells. Data represent three (**a**,**b**,**e**) or two (**c**,**d**,**f**,**g**) independent experiments (*n*=2 per group per experiment). Data are presented as means±s.e.m. Not Significant (NS) *P*>0.05, *P<0.05, **P<0.01 determined by Student's *t*-test.

We further analysed phenotypic characteristics of splenic J15 CD8^+^ T cells after helper-deficient priming. These cells upregulated CD69 and CD44, produced IL-2 and IFN-γ and expressed CXCR3 (necessary for egress to the peripheral tissues)^35^ and CD122 (IL-2Rβ and IL-15Rβ) after four to five cell divisions, similar to helped cells (Fig. 7a and Supplementary Fig. 4a). Analysis of cells from FTY-720-treated mice confirmed intact differentiation of the helper-deficient CD8^+^ T cells into functional effectors expressing normal levels of CXCR3, CD122 and granzyme B (Fig. 7b). Moreover, no significant differences were observed in Annexin-V staining or Bcl-2 expression between helper-deficient and helped cells (Supplementary Fig. 4b). However, helper-deficient T cells analysed on day 7 post priming expressed CD69 with early CFSE-dilution peaks (Fig. 7a), indicating initiation of a new wave of activation immediately following first burst expansion of helper-deficient T cells. These patterns of intact effector differentiation, as well as fast response kinetics, of helper-deficient CD8^+^ T cells were further verified by analysis of J15 CD8^+^ T cells transferred in low numbers or polyclonal H60-tetramer-binding CD8^+^ T cells in helper-deficient hosts (Fig. 7c,d and Supplementary Fig. 4c,d).

Effector differentiation of helper-deficient T cells with advanced kinetics was also seen when J15 CD8^+^ T cells were activated *in vitro* without Marylin TCR Tg CD4 T cells specific for Hy-Dby/H-2A^b^ or when OT-1 T cells in adoptive hosts were primed by injection of spleen cells from ovalbumin (Ova)-Tg mice, which express membrane-bound chicken Ova under the control of the beta-actin promoter^36^, with concurrent CD4 depletion (Supplementary Fig. 4e,f). Moreover, helper-deficient OT-1 cells exhibited newly activated cells on day 7 post priming. Altogether, the phenotypic characteristics of helper-deficient CD8^+^ T cells in the expansion phase revealed that they had undergone conventional activation and effector differentiation, although newly activated cells reappeared on day 7.

Then, we compared helper-deficient and helped J15 CD8^+^ T cells with respect to their effector fate decision into short-lived effector cells (SLECs; CD127^low^KLRG-1^hi^) and memory precursor effector cells (MPECs; CD127^hi^KLRG-1^low^), which become terminally differentiated effectors and long-term memory cells, respectively^37^. We found that helper-deficient T cells generated MPECs and SLECs, as did helped cells (Fig. 7e). Overall trends of the changes in the proportions of the SLEC and MPEC populations were similar between helper-deficient and helped cells, as the MPEC population increased gradually over time. The peak of the SLEC population was 2 days earlier (day 5) with a much lower number in helper-deficient hosts. The ability of helper-deficient CD8^+^ T cells to generate MPECs was further verified using J15 CD8^+^ T cells transferred in low numbers (Fig. 7f and Supplementary Fig. 5) and polyclonal H60-tetramer-binding CD8^+^ T cells on day 10 post priming (Fig. 7g).

### CD8^+^ T_cm_ generation without CD4 help by antigen clearance

**Figure 8:**
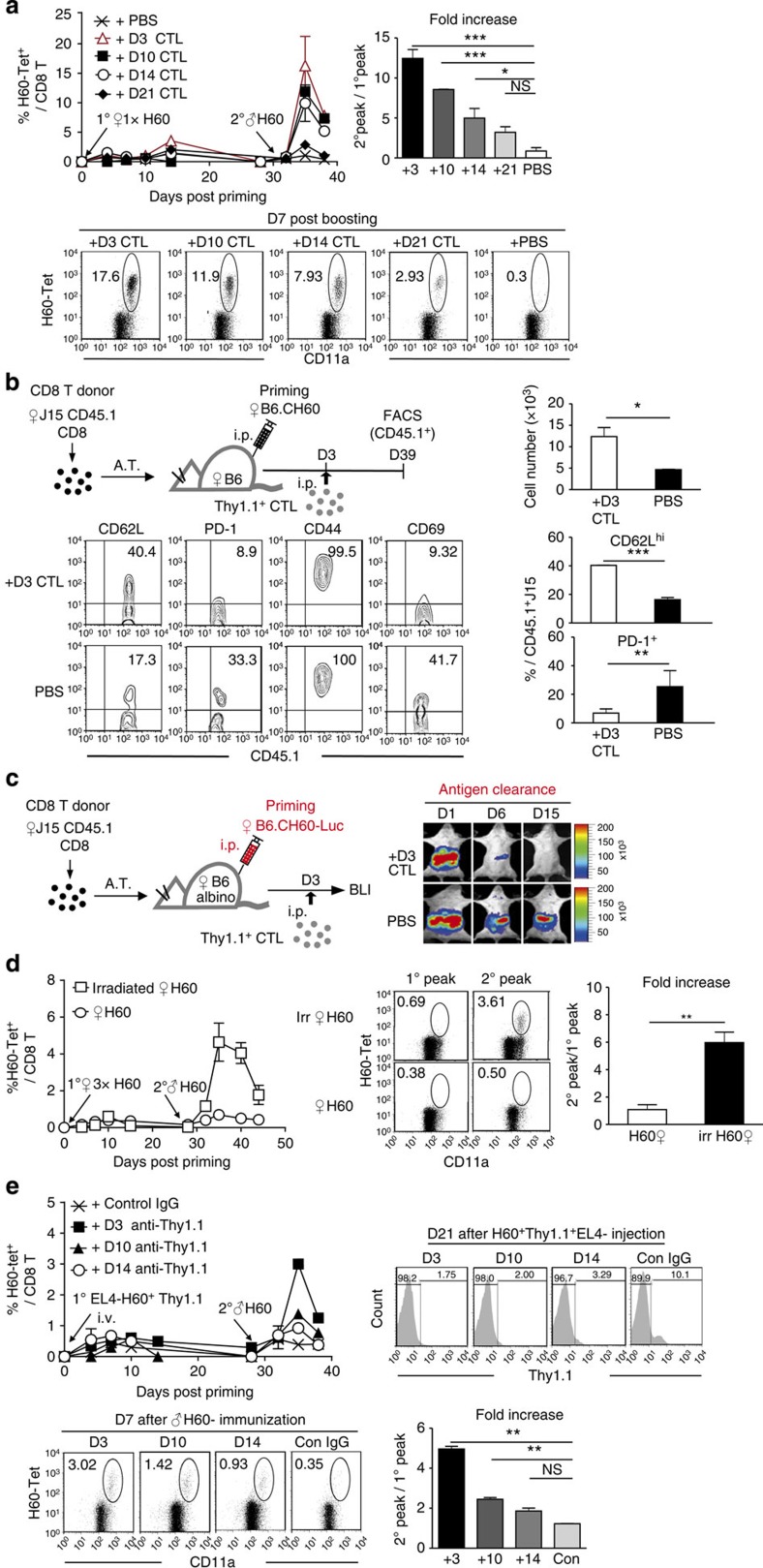
Enhanced T_cm_ differentiation and memory expansion of helper-deficient H60-specific CD8^+^ T cells by early antigen clearance. (**a**) Longitudinal PBL analysis of helper-deficient B6 mice (Thy1.1^−^) treated i.p. with H60-specific Thy1.1^+^ CTLs (1 × 10^6^) at the indicated time points and boosted on day 30 post priming. Plots of the frequency of H60-tetramer-binding cells after gating of Thy1.1^−^ CD8^+^ cells; the fold increases in peak values (day 7 post boosting versus day 10–14 post priming); and representative flow cytometric data (day 7 post boosting) are shown. (**b**) Phenotypic analysis of CD45.1^+^ J15 CD8^+^ T cells in helper-deficient hosts with day-3-CTL treatment on day 39 post priming. Numbers, representative flow cytometric data and plots of the percentages of CD62L^hi^ and PD-1^+^ cells in the CD45.1^+^ J15 CD8^+^ T-cell population are presented. (**c**) Longitudinal BLI analysis of injected female B6.CH60-LucTg cells. (**d**) Longitudinal PBL analyses of female B6 mice primed with lethally irradiated (2,000 cGy) female B6.CH60 splenocytes. Memory expansion of H60-specific CD8^+^ T cells after boosting with male B6.CH60 splenocytes was compared with that in helper-deficient mice primed with live B6.CH60 splenocytes. Flow cytometric data on day 10 post primary (1^o^ peak) and day 7 post boosting (2^o^ peak) are shown. The fold increases in the frequencies of H60-tetramer-binding CD8^+^ T cells are plotted. (**e**) Longitudinal PBL analyses of female B6 mice injected i.v. with H60^+^ Thy1.1^+^EL-4 tumour cells. The tumour-injected mice were treated with anti-Thy1.1 antibody or control rat anti-mouse IgG at the indicated time points, and then boosted with male B6.CH60 splenocytes on day-40 post-tumour injection. Flow cytometric data after staining the PBL with anti-Thy1.1 mAb on day 21 post-tumour injection are shown. Flow cytometric data showing the H60-tetramer-positive CD8^+^ T cells on day 7 post boosting (2^o^ peak) and plots of fold increases in the frequencies of H60-tetramer-binding CD8^+^ T cells on day 7 post boosting relative to those on day 7–10 post-tumour injection (1^o^ peak) are presented. All data (**a–e**) are representative of two independent experiments (*n*=2–3 per group per experiment). A.T., Adoptive Transfer. Data are presented as means±s.e.m. Not Significant (NS) *P*>0.05, *P<0.05, **P<0.01, ***P<0.001 determined by Student's *t*-test.

Since helper-deficient CD8^+^ T cells possessed effector/memory differentiation capacity, the main differences between helper-deficient and helped conditions were low effector burst size during the expansion phase (Fig. 5) and delayed overall antigen clearance (Fig. 2). Therefore, we examined whether deliberate elimination of the H60 allografts could rescue helper-deficient CD8^+^ T cells from exhaustion and promote their memory expansion. We injected 1 × helper-deficient B6 mice (Thy1.1^−^) with *in vitro* established H60-specific CTL lines (Thy1.1^+^) to kill the H60 allografts (to clear antigen) on days 3, 10, 14 or 21, enabling an evaluation of the influence of antigen duration on the fate of H60-specific CD8^+^ T cells. Longitudinal flow cytometric PBL analyses after gating for host Thy1.1^−^ CD8^+^ cells showed that the earlier CTLs were treated, the greater was the expansion of the helper-deficient T cells after boosting (days 3>10>14>21; Fig. 8a), indicating a correlation between the timing of antigen clearance and the degree of memory expansion.

A phenotypic analysis of the J15 CD8^+^ T cells from helper-deficient hosts treated on day 3 with CTLs confirmed increased T_cm_ generation by the CTL treatment on day 39 post priming (Fig. 8b). A BLI analysis of primed H60 allografts verified accelerated antigen clearance in helper-deficient mice by the day-3-CTL treatment (Fig. 8c). Thus, shortening the length of exposure to antigen facilitated differentiation of helper-deficient CD8^+^ T cells into T_cm_. Consistently, priming with lethally irradiated female B6.CH60 splenocytes instead of live ones enhanced memory generation by the helper-deficient CD8^+^ T cells specific for H60 (Fig. 8d). It was unlikely that this memory enhancement in helper-deficient hosts was caused by inflammation in itself provoked during priming with irradiated splenocytes because deliberate induction of inflammation by injecting TLR ligands (Pam3CSK4, LPS or loxoribine) could not rescue helper-deficient CD8 T cells for H60 from the memory impairment (Supplementary Fig. 6), and because such memory enhancement was not observed in the helped hosts primed with irradiated male B6.CH60 splenocytes (Supplementary Fig. 7). Moreover, supplementation of IL-2 into helper-deficient hosts at various time points after priming did not elicit memory expansion of the H60-specific CD8^+^ T cells (Supplementary Fig. 8). In addition, antibody-mediated killing of H60-positive tumours at early time points enhanced memory expansion of the helper-deficient CD8^+^ T cells for H60 (Fig. 8e), illustrating the enhancement of memory cell generation by helper-deficient CD8^+^ T cells through early clearance of antigen, regardless of CTL supplementation. Taken altogether, these results suggest that the requirement for CD4^+^ T-helper cells could be bypassed by early clearance of the antigen.

### T_cm_ generation by adding helper-deficient CD8^+^ effectors

**Figure 9:**
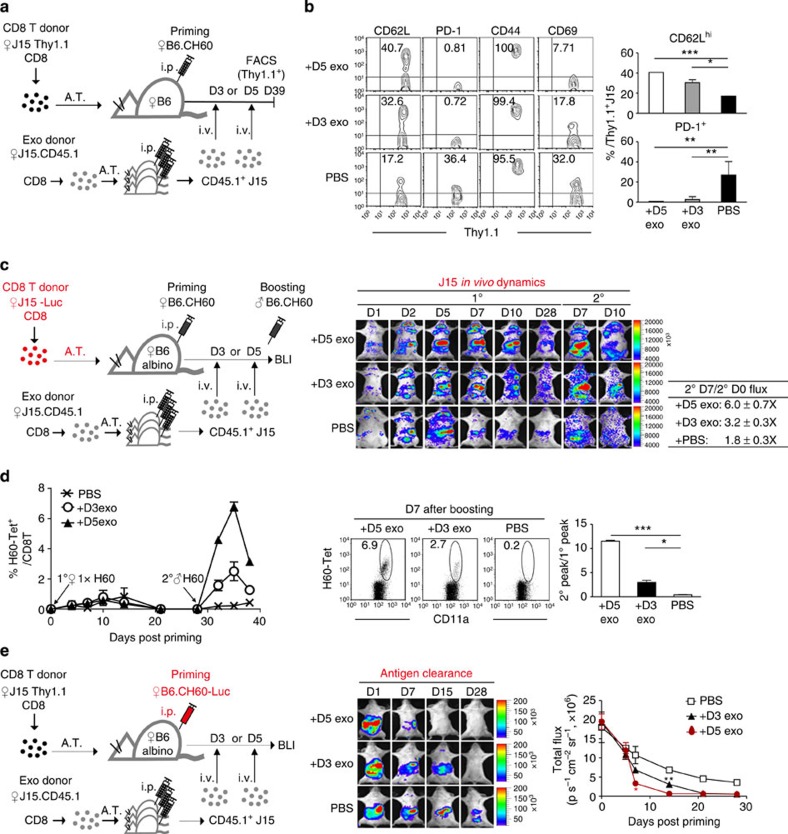
Memory generation under helper-deficient conditions by supplementation with helper-deficient CD8^+^ T cells at the effector stage. (**a**) Two groups of adoptive hosts (one transferred with Thy1.1^+^ J15 cells, and the other with CD45.1^+^ J15 cells) were primed under helper-deficient conditions on the same day. On day 3 or 5 post priming, CD45.1^+^ J15 CD8^+^ T cells were purified from three helper-deficient hosts. These cells or PBS (on day 5) were injected i.v. (1 × 10^6^) on day 5 into the other adoptive helper-deficient host. Phenotyping was performed on day 39 post priming. (**b**) Flow cytometric data and plots of the proportions of CD62^hi^ or PD-1^+^ cells in Thy1.1^+^ J15 CD8^+^ T cells. (**c**) Longitudinal BLI analysis of endogenous J15-LucTg CD8^+^ T cells in the recipients. (**d**) Exogenous helper-deficient CD45.1^+^ J15 CD8^+^ T cells or PBS were injected i.v. into helper-deficient B6 mice, which were then boosted on day 30 post priming. The frequency of H60-tetramer-binding CD8^+^ T cells after gating of CD45.1^−^ CD8^+^cells are presented. Representative flow cytometric data (day 7 post boosting) and a plot of the fold increases in peak values (day 7 post boosting versus day 10 post priming) are shown. (**e**) Longitudinal BLI analysis of injected female B6.CH60-LucTg cells. Data are representative of two (**c**,**e**) or three (**b**,**d**) independent experiments (*n*=2–3 per group per experiment). A.T., Adoptive Transfer. Data are presented as means±s.e.m. *P<0.05, **P<0.01, ***P<0.001 determined by Student's *t*-test.

We hypothesized that increasing the size of the burst effector during the helper-deficient response could mimic the presence of CD4^+^ T-helper cells. Thus, we prepared additional effector J15 cells (CD45.1^+^) from another set of mice separately primed under helper-deficient conditions. CD45.1^+^ J15 CD8^+^ T cells purified from three helper-deficient hosts in this set were injected intravenously (1.5–2 × 10^6^ cells per mouse) into a CD45.1^−^ helper-deficient adoptive host in another group on days 3 or 5 post priming, which was transferred with Thy1.1^+^ J15 cells (Fig. 9a). This manipulation increased the size of the effector pool at the premature (D3) or the matured (D5) effector stage in the helper-deficient response (Fig. 7a), respectively.

Phenotyping of Thy1.1^+^ J15 CD8^+^ T cells from the recipients of helper-deficient CD45.1^+^ cells or control PBS on day 39 post priming demonstrated that the proportions of CD62L^hi^, PD-1^−^ and CD69^−^ populations (in T_cm_ status) in J15 Thy1.1^+^ CD8^+^ T cells increased significantly after supplementation with exogenous helper-deficient T cells, compared with the PBS-supplemented control group (Fig. 9b). In particular, the proportion of T_cm_ cells increased to a level normally observed in helped cells (Fig. 3c) after supplementation with day-5 cells. Appropriate localization of the injected helper-deficient T cells (J15-LucTg CD8^+^ T cells) in the spleen, LNs and peritoneum of the helper-deficient recipients was confirmed by BLI analysis (Supplementary Fig. 9). A BLI analysis using J15-LucTg cells as endogenous helper-deficient T cells spatiotemporally confirmed their differentiation into memory cells, showing enhanced localization in the LNs and five- to sevenfold increased cell expansion after boosting the recipients of day-5 cells (Fig. 9c).

To determine whether the changes after transfer of J15 CD8^+^ T cells could be reproduced using polyclonal H60-specific CD8^+^ T cells, 1 × helper-deficient B6 mice were supplemented with helper-deficient J15 CD8^+^ T cells (CD45.1^+^) as above. Longitudinal flow cytometric PBL analysis after gating the CD45.1^−^ CD8^+^ cells of recipient origin demonstrated that the peak frequencies of H60-tetramer-binding CD8^+^ T cells increased threefold on day-3 supplementation or 11-fold on day-5 supplementation after boosting compared with the primary peaks in the recipients (Fig. 9d). In addition, a BLI analysis of the H60 allografts (B6.CH60-LucTg cells) demonstrated that antigen clearance was accelerated significantly in the exogenous cell recipients by the day-5 supplementation (Fig. 9e).

Combined with the data shown in Fig. 8, these results revealed that helper-deficient CD8^+^ T cells were not predestined to become T_em_ (exhausted or tolerized); increasing the number of cells at the mature effector stage was sufficient to stimulate differentiation into T_cm_ and facilitate antigen clearance, suggesting that the memory generation programme is intrinsic to CD8^+^ T cells and is not encoded by CD4^+^ T-helper cells.

## Discussion

In this study, we showed that impaired memory generation by CD8^+^ T cells activated in the absence of CD4 help is due to a failure to generate sufficient numbers of effector CD8 T cells at initial burst expansion on priming and the subsequent failure of efficient antigen clearance, and also that these T cells become eventually exhausted due to antigen persistence in our model. Most importantly, we showed that helper-deficient CD8^+^ T cells retain the ability to generate T_cm_.

The role of CD4^+^ T-helper cells in the CD8^+^ T-cell response has not been entirely understood. Using minor histocompatibility antigen H60 as a single antigen generating CD8 epitope, we investigated the effects of CD4 help on CD8^+^ T-cell response, choosing to characterize the fate of CD8^+^ T cells activated without CD4 help instead of focusing on CD4^+^ T cells themselves. Other than CD4 help, several CD8^+^ T-cell-intrinsic factors such as TCR affinity, precursor frequency and the timing of CD8^+^ T-cell recruitment to the response have been suggested as fate-determining factors^38^,^39^,^40^. In our J15 system, CD4 help appeared to be a major fate-determining factor, since other CD8^+^ T-cell-intrinsic factors such as TCR affinity and precursor frequency were fixed, and deprivation of CD4 help impaired memory CD8^+^ T-cell generation. However, surprisingly, in the absence of CD4 help, elevating ratios of CD8^+^ effector number to antigenic load (T_eff_/Ag) through either early antigen clearance (Fig. 8) or augmented CD8^+^ effector burst size (Fig. 9) favoured T_cm_ generation. That is, lowering antigenic load on CD8^+^ effectors favoured memory cell generation, irrespective of CD4 help, while elevating antigenic dose (3 × ) intensified exhaustion of helper-deficient CD8^+^ T cells. Other studies using infectious models have also shown inverse correlation between degree of memory expansion and antigen abundance during chronic infection^41^ and promotion of memory CD8^+^ T-cell generation with limited exposure to infection^38^,^42^,^43^. Thus, the T_eff_/Ag ratio affects fates of CD8^+^ T cells in the memory phase. In addition, fate of CD8^+^ T to become T_cm_ depends on antigen clearance, rather than on the presence of CD4 help. Furthermore, the fact that CD8^+^ T cells could differentiate into long-term memory precursors after helper-deficient priming implies presence of CD8^+^ T-cell-intrinsic activation and differentiation programme, without CD4 help. We, therefore, suggest that the memory differentiation programme is CD8^+^ T-cell-intrinsic, not encoded by CD4 help. Instead, CD4^+^ T cells may help CD8^+^ T cells sustain the intrinsic memory programme, providing them direct quantitative benefits for the expansion and, hence, efficient antigen clearance.

We demonstrated that tolerance of alloantigen-specific CD8^+^ T cells after helper-deficient priming corresponds to CD8^+^ T-cell exhaustion in our model. Naive and antigen-experienced CD8^+^ T cells are found to be continuously recruited in response from initial priming through the memory phase (Fig. 3). Repeated re-activation of exhausted T cells may cause their eventual elimination^41^, thereby shrinking the size of the entire pool of antigen-reactive T cells. However, continuous recruitment of naive J15 CD8^+^ T cells for activation may maintain the exhausted pool until the end of the helper-deficient response^44^. Therefore, we suggest that continuous antigen encounters by the specific CD8^+^ T cells because of antigen persistence may prevent their becoming functional memory cells, with the exhausted CD8^+^ T cells remaining in memory phase being tolerant to antigen rechallenge, as supported by restored memory generation with PD-1 blockade (Fig. 4).

It has been reported that CD8^+^ T cells activated in the absence of CD4 help were defective in cytotoxic activity^12^,^13^,^14^, do not express CD25, CD122, IFN-γ and granzyme B^45^,^46^ and do not migrate to peripheral sites^47^, suggesting that helper-deficient CD8^+^ T cells would be functionally abortive and qualitatively different from helped cells. In contrast, we demonstrated that they are functionally normal after being primed, expressing activation and effector molecules properly, and are able to migrate to peripheral antigen sites and differentiate into SLECs and MPECs. Our results are consistent with previous reports that CD8^+^ T cells activated in CD4-depleted or MHC II- or CD4-deficient mice could express IFN-γ and express cytotoxic activity^2^,^3^,^4^, although they did not study the helper-deficient CD8^+^ T cells in a response kinetics context, nor elucidate how they become impaired in generating memory cells. Helper-deficient cells differed only in their response kinetics, which is characterized as an early egress to the periphery after shortened and insignificant burst expansion or lack of extended and massive expansion within the secondary lymphoid organs. The discrepancies between our results and the previous reports could be due to differences in tools used and in the time points studied to characterize the helper-deficient cells. The first ever detailed longitudinal BLI and flow cytometric analyses of helper-deficient CD8^+^ T cells, which we report here, provide precise spatiotemporal information precisely regarding the *in vivo* status of H60-specific CD8^+^ T cells and antigens.

This study elucidates the cellular mechanism underlying the memory impairment of CD8^+^ T cells activated without CD4 help and the importance of antigen clearance in CD8^+^ T_cm_ generation, suggesting the presence of CD8^+^ T-cell-intrinsic memory programme and quantitative role of CD4 help. The advantage of system employed is that it exploited an endogenous cellular antigen, and controlled CD4 help under physiological conditions. Cell-based antigens are considered to be non-inflammatory and induce CD8^+^ T-cell response in a CD4 help-dependent manner, requiring the presence of CD4 help for primary expansion of the specific CD8^+^ T cells. However, CD8^+^ T-cell responses to infectious pathogens are different, in that infections generally incur severe inflammation and T-cell activation and in that some of these antigens actually can induce the primary expansion of the specific CD8^+^ T cells without CD4 help. Thus, extending our suggestions to other model systems in terms of quantitative aspects of CD4 help may be limited and will require further studies on the dynamics of CD8^+^ T cells primed against infectious antigens without CD4 help. Nonetheless, we suggest that this study provides insights into how CD4 help and CD8^+^ T-cell immunity can be used to design strategies to enhance CD8^+^ T-cell memory for antitumour immunity or to promote transplantation tolerance.

## Methods

### Mouse lines

C57BL/6 (B6), C.B10-*H2*^*b*^/LiMcdJ (BALB.B), B6.SJL-*Ptprc*^*a*^*Pep3*^*b*^/BoyJ (CD45.1^+^), B6.PL-*Thy1*^*a*^/CyJ (Thy1.1^+^), B6-Tg(TcraTcrb)1100Mjb/J (OT-1), B6-Tg(CAG-OVA)916Jen/J (B6-OVATg), B6.129P2-*B2m*^*tm1Unc*^ (*β2m*^−/−^), B6.129S2-*Pdcd1*^tm1Hon^/HonRbrc (*Pdcd1*^−/−^), B6(Cg)-*Tyr*^*c-2J*^/J (B6-Albino) and B6.C-*H60*^*c*^/DCR (B6.CH60) mice were obtained from the Jackson Laboratory (Bar Harbor, ME, USA). Transgenic luciferase mice (B6-Tg[CAG-effLuc]; B6-LucTg) described previously^26^ were backcrossed to B6.CH60 mice. TCR transgenic mouse lines with TCR specific to the H60-CD8 epitope/H-2K^b^ were generated after microinjection of a fertilized B6 egg with eukaryotic DNA fragments from pTα and pTβ cassette plasmids^48^ containing TCRV_α_-J_α_ and TCRV_β_-D_β_-J_β_ rearranged genomic sequences originating from H60-specific CTL clone 15 (ref. 27), respectively. The transgenic mice were generated by injection of linearized DNA fragments devoid of prokaryotic sequences into fertilized eggs of B6 mice. Transgenic founders were identified by PCR analysis of genomic DNA and flow cytometric analysis of PBLs after staining with anti-V_β_8.3 antibody. Among the three founder lines showing positive selection on a B6 background and negative selection on a B6.CH60, we selected one line (#15; designated J15) in which the TCR levels on peripheral blood CD8^+^ T cells were comparable to those of wild-type B6 T cells (Supplementary Fig. 2). The J15 line was backcrossed into a CD45.1^+^, Thy1.1^+^, or *Pdcd1*^−/−^ background or crossed with B6-LucTg (J15-LucTg). *β2m*^−/−^ mice were backcrossed to a H60 congenic background to generate *β2m*^−/−^ B6.CH60 mice.

All mice were maintained under specific pathogen-free conditions at the Center for Animal Resource Development of Seoul National University, College of Medicine, Korea and used for experiments at ages of 8–12 weeks with the approval of the Institutional Animal Care and Use Committee of Seoul National University.

### Adoptive transfer and immunization

CD8^+^ T cells from CD45.1^+^J15, J15-LucTg or *Pdcd1*^−/−^ J15 mice were purified from the spleen and LNs by negative magnetic-activated cell sorting (Miltenyi Biotec, Auburn, CA, USA). After labelling with CFSE (eBioscience, San Diego, CA, USA), cells were adoptively transferred (1–2 × 10^6^ or 0.1–1 × 10^4^) 1 day before priming. A single-cell suspension (2 × 10^7^ in 200 μl PBS) of splenocytes from B6.CH60 mice was injected i.p. to induce an H60-specific CD8^+^ T-cell response. FTY-720 (0.3 mg kg^−1^; Biovision, Milpitas, CA, USA) was injected i.p. daily from day 2 post priming until the mice were euthanized.

### Antibody treatment for cell-depletion and blockade

Mice were injected i.p. with ascites fluid of anti-mouse CD4 monoclonal antibody (GK1.5) at 1 and 3 days before immunization. Rat anti-mouse PD-L1 antibody (200 μg per mouse; 10 F:9G2; Bio X Cells, West Lebanon, NH, USA) or rat IgG2b isotype control (Bio X Cells) were administered i.p. five times every 3 days beginning on day 14 after immunization for PD-L1 blockade. Female B6 mice intravenous (i.v.) injected with EL-4 tumour cells (2 × 10^5^) transduced to co-express H60 and Thy1.1 were treated i.p. with anti-Thy1.1 antibody (200 μg per mouse; 19E12; Bio X cells) or control rat anti-mouse IgG (Bio X cells) for two consecutive days starting at various time points after tumour injection.

### Microbead-based enrichment of H60-specific CD8^+^ T cells

Magnetic bead-based enrichment of T cells was performed as described previously^29^. To track the low number of CD45.1^+^ J15 cells, single-cell suspensions of splenocytes prepared from adoptive hosts were stained with biotin-conjugated anti-CD45.1 (identified above), and then washed and stained with anti-biotin magnetic microbeads (Miltenyi Biotec) followed by enrichment via a magnetic column. Splenocytes from primed B6 mice (without J15 transfer) were stained with primary phycoerythrin (PE)-conjugated H60 tetramer and anti-PE magnetic microbeads to track the polyclonal H60-tetramer-binding CD8^+^ T cells.

### Antibodies and flow cytometry

Single-cell suspensions were stained with antibodies or H60 tetramers (LTFNYRNL/H-2K^b^) at 4 °C for 30 min in staining buffer (1 × PBS containing 0.1% bovine serum albumin and 0.1% sodium azide). Intracellular staining was performed after cell fixation and permeabilization^22^. Cells were analysed using a FACSCalibur (BD Pharmingen, San Diego, CA, USA) or LSRII-Green (BD Pharmingen) and data were analysed using the FlowJo software (Tree Star, Ashland, OR, USA). Antibodies used for flow cytometric analysis were as follows. Fluorescent-dye-conjugated antibodies against CD8 (1:1,000; 53–6.7), CD40 (1:500; 1C10), CD44 (1:2,000; IM7), CD45.1 (1:1,000; A20), CD62L (1:2,000; MEL-14), CD80 (1:1,000; 16-10A1), CD86 (1:1,000; GL1), Thy1.1 (1:1,000; HIS51), CD122 (1:1,000; TM-b1), CD127 (1:1,000; A7R34), H-2K^b^ (1:500; AF6-88.5), mIgG (1:600; M1-14D12), granzyme B (1:4,000; 16G6), IFN-γ (1:2,000; XMG1.2), IL-2 (1:1,000; JES6-5H4) and CXCR3 (1:1,000; CXCR3-173) were all purchased from eBioscience. Anti-CD11a (1:1,000; 2D7), -PD-1 (1:1,000; 29 F.1A12) and -KLRG1 (1:1,000; 2F1) antibodies were purchased from BD Pharmingen. Anti-CD69 antibody (1:1,000; H1.2F3) was purchased from BioLegend (San Diego, CA, USA), and anti-Blimp-1 antibody (1:500; N-20) was from Santa Cruz Biotechnology (Dallas, TX, USA).

### Establishment and injection of CTL lines

Thy1.1^+^H60-specific CTL lines were established as described previously^17^. In brief, female Thy1.1^+^ mice were injected i.p. with 2 × 10^7^ splenocytes from male H60 congenic mice (B6.CH60). Splenic CD8^+^ T cells were harvested from the immunized mice on day 7 and cultured *ex vivo* with irradiated male H60 congenic splenocyte feeder cells in the presence of recombinant hIL-2 (50 U ml^−l^; Sigma-Aldrich) in DMEM media containing 10% FBS (HyClone Laboratories, Logan, UT, USA) and antibiotics. They were maintained by weekly restimulation with irradiated feeder cells. During the 7-day culture period, CD8^+^ T cells underwent activation and resting cycles. The status of the CTL lines was monitored via flow cytometry to maintain the purity of H60-tetramer-binding CD8^+^ T cells at >99%. CTLs on day 5 after restimulation (2 × 10^6^ cells) were washed twice with 1 × PBS before i.p. injection.

### *In vivo* BLI

*In vivo* BLI was performed using an IVIS 100 imaging system with a charge-coupled device camera (Caliper Life Sciences, Hopkinton, MA, USA) as described previously^26^. Mice were kept on stage under anaesthesia (1.5% isoflurane gas in oxygen at a flow rate of 1.5 l min^−1^) and i.p. injected with D-luciferin (150 mg kg^−1^; Molecular Probes, Eugene, Oregon, USA). Mice were positioned supine to image ventral surfaces. Relative intensities of emitted light were pseudocoloured, ranging from red (most intense) to blue (least intense). Grey-scale photographs and the corresponding pseudocolour images were superimposed with the LIVINGIMAGE (ver2.12; Xenogen, Alameda, CA, USA) and IGOR (WaveMetrics, Oswego, OR, USA) software. Signals emitted by regions of interest were expressed as photon flux (photon s^−1^ cm^−2^ steradian^−1^ (sr^−1^)), which refers to the photons emitted from a unit solid angle of a sphere.

### *In vivo* cytotoxicity assay

B6 splenocytes labelled with 2.5 μM CFSE were pulsed with H60 peptide, while those with 0.42 μM CFSE were pulsed with control VSV peptide. Then, mixtures (1:1) of the CFSE-labelled and peptide-loaded target cells were i.v. injected into tolerized-and-boosted or helped-and-boosted hosts on day 7 post boosting, or naive control mice. Flow cytometry was performed 72 h later to analyse PBLs from the injected mice and to detect the CFSE-labelled cells.

### *In vitro* IFN-γ production assay

Splenocytes harvested from tolerized-and-boosted or helped-and-boosted hosts on day 7 post boosting were *in vitro* stimulated with H60 or VSV control peptide, fixed and permeabilized for staining with anti-IFN-γ antibody and subsequent flow cytometric analysis^22^.

### RNA isolation and quantitative RT–PCR

Total RNA was extracted using TRIzol reagent according to the manufacturer's instructions (Invitrogen, Carlsbad, CA, USA). cDNA was synthesized for each template with total RNA incubated at 42 °C for 80 min with a mixture of 200 units M-MLV reverse transcriptase (Takara Bio, Otsu, Shiga, Japan), 1 × RT buffer, 0.25 mM dNTP and 40 units RNase inhibitor (Koschem Co., Seoul, Korea). Quantitative PCR was performed as described by the manufacturer of SYBR premix ExTaq (Takara Bio) in 96-well plates on a thermal cycler (Light Cycler 96; Roche, Mannheim, Germany). β-actin mRNA levels were used for normalization. The oligonucleotide sequences used for qPCR were as follows: *Pdcd1* forward, 5′-CCGCCTTCTGTAATGGTTTGA-3′; *Pdcd1* reverse, 5′-GGGCAGCTGTATGATCTGGAA-3′; *Sell* forward, 5′-CTCGAGGAACATCCTGAAGC-3′; *Sell* reverse 5′-AGCATTTTCCCAGTTCATGG-3′; *Prdm1* forward, 5′-AAGCTCAAGAAAGGAAACATGC-3'; *Prdm1* reverse, 5′-TGGGTTGCTTTCCGTTTG-3′; *Tim-3* forward 5′-CGGAGAGAAATGGTTCAGAGACA-3′; *Tim-3* reverse, 5′-TTCATCAGCCCATGTGGAAAT-3′; *Lag3* forward, 5′-TCACTGTTCTGGGTCTGGAG-3′; *Lag3* reverse, 5′-CACTTGGCAGTGAGGAAAGA-3′. *b-actin* forward, 5′-GGCTGTATTCCCCTCCATCG-3′; *b-actin* reverse, 5′-CCAGTTGGTAACAATGCCATGT-3′.

### Statistical analysis

Statistical analysis was performed using GraphPad Prism ver. 5 (GraphPad Software, San Diego, CA, USA). Data are presented as the means± s.e.m. *P* values were determined by Student's *t*-tests with *P<0.05, **P<0.01, ***P<0.001. A *P* value<0.05 considered to indicate statistical significance.

## Additional information

**How to cite this article:** Kim, J. *et al.* Memory programming in CD8^+^ T-cell differentiation is intrinsic and is not determined by CD4 help. *Nat. Commun.* 6:7994 doi: 10.1038/ncomms8994 (2015).

## Supplementary Material

Supplementary Information Supplementary Figures 1-9

## References

[b1] Bevan M. J. Helping the CD8^+^ T-cell response. Nat. Rev. Immunol. 4, 595–602 (2004). 15286726 10.1038/nri1413

[b2] Janssen E. M. *et al.* CD4^+^ T cells are required for secondary expansion and memory in CD8^+^ T lymphocytes. Nature 421, 852–856 (2003). 12594515 10.1038/nature01441

[b3] Shedlock D. J. & Shen H. Requirement for CD4 T cell help in generating functional CD8 T cell memory. Science 300, 337–339 (2003). 12690201 10.1126/science.1082305

[b4] Sun J. C. & Bevan M. J. Defective CD8 T cell memory following acute infection without CD4 T cell help. Science 300, 339–342 (2003). 12690202 10.1126/science.1083317 PMC2778341

[b5] Schoenberger S. P., Toes R. E., van der Voort E. I., Offringa R. & Melief C. J. T-cell help for cytotoxic T lymphocytes is mediated by CD40-CD40L interactions. Nature 393, 480–483 (1998). 9624005 10.1038/31002

[b6] Huster K. M. *et al.* Selective expression of IL-7 receptor on memory T cells identifies early CD40L-dependent generation of distinct CD8^+^ memory T cell subsets. Proc. Natl Acad. Sci. USA 101, 5610–5615 (2004). 15044705 10.1073/pnas.0308054101 PMC397444

[b7] Castellino F. & Germain R. N. Cooperation between CD4^+^ and CD8^+^ T cells: when, where, and how. Ann. Rev. Immunol 24, 519–540 (2006). 16551258 10.1146/annurev.immunol.23.021704.115825

[b8] Schluns K. S. & Lefrancois L. Cytokine control of memory T-cell development and survival. Nat. Rev. Immunol. 3, 269–279 (2003). 12669018 10.1038/nri1052

[b9] Carr J. M. *et al.* CD27 mediates interleukin-2-independent clonal expansion of the CD8^+^ T cell without effector differentiation. Proc. Natl Acad. Sci. USA 103, 19454–19459 (2006). 17159138 10.1073/pnas.0609706104 PMC1697827

[b10] Peperzak V., Xiao Y., Veraar E. A. & Borst J. CD27 sustains survival of CTLs in virus-infected nonlymphoid tissue in mice by inducing autocrine IL-2 production. J. Clin. Invest. 120, 168–178 (2010). 19955658 10.1172/JCI40178 PMC2798690

[b11] Peperzak V. *et al.* CD8^+^ T cells produce the chemokine CXCL10 in response to CD27/CD70 costimulation to promote generation of the CD8^+^ effector T cell pool. J. Immunol. 191, 3025–3036 (2013). 23940275 10.4049/jimmunol.1202222

[b12] Antoniou A. *et al.* T cell tolerance and activation to a transgene-encoded tumor antigen. Eur. J. Immunol. 26, 1094–1102 (1996). 8647173 10.1002/eji.1830260521

[b13] VanderVegt F. P. & Johnson L. L. Induction of long-term H-Y-specific tolerance in female mice given male lymphoid cells while transiently depleted of CD4^+^ or CD8^+^ T cells. J. Exp. Med. 177, 1587–1592 (1993). 8098730 10.1084/jem.177.6.1587 PMC2191056

[b14] Filatenkov A. A. *et al.* CD4 T cell-dependent conditioning of dendritic cells to produce IL-12 results in CD8-mediated graft rejection and avoidance of tolerance. J. Immunol. 174, 6909–6917 (2005). 15905533 10.4049/jimmunol.174.11.6909

[b15] Zhai Y., Wang Y., Wu Z. & Kupiec-Weglinski J. W. Defective alloreactive CD8 T cell function and memory response in allograft recipients in the absence of CD4 help. J. Immunol. 179, 4529–4534 (2007). 17878349 10.4049/jimmunol.179.7.4529

[b16] Roopenian D., Choi E. Y. & Brown A. The immunogenomics of minor histocompatibility antigens. Immunol. Rev. 190, 86–94 (2002). 12493008 10.1034/j.1600-065x.2002.19007.x

[b17] Choi E. Y. *et al.* Quantitative analysis of the immune response to mouse non-MHC transplantation antigens *in vivo*: the H60 histocompatibility antigen dominates over all others. J. Immunol. 166, 4370–4379 (2001). 11254691 10.4049/jimmunol.166.7.4370

[b18] Choi E. Y. *et al.* Immunodominance of H60 is caused by an abnormally high precursor T cell pool directed against its unique minor histocompatibility antigen peptide. Immunity 17, 593–603 (2002). 12433366 10.1016/s1074-7613(02)00428-4

[b19] Malarkannan S. *et al.* The molecular and functional characterization of a dominant minor H antigen, H60. J. Immunol. 161, 3501–3509 (1998). 9759870

[b20] Scott D. *et al.* Dendritic cells permit identification of genes encoding MHC class II-restricted epitopes of transplantation antigens. Immunity 12, 711–720 (2000). 10894170 10.1016/s1074-7613(00)80221-6

[b21] Jung K. M. & Choi E. Y. Role for CD40 and CD40L expression in generating CD8 T cell response to minor histocompatibility antigen, H60. Immune Netw. 7, 6 (2007).

[b22] Ryu S. J. *et al.* Cognate CD4 help is essential for the reactivation and expansion of CD8 memory T cells directed against the hematopoietic cell-specific dominant minor histocompatibility antigen, H60. Blood 113, 4273–4280 (2009). 19139082 10.1182/blood-2008-09-181263

[b23] Choi J. H. *et al.* TCR diversity of H60-specific CD8 T cells during the response evolution and influence of CD4 help. Transplantation 87, 1609–1616 (2009). 19502951 10.1097/TP.0b013e3181a52dc4

[b24] Alexander M. A., Damico C. A., Wieties K. M., Hansen T. H. & Connolly J. M. Correlation between CD8 dependency and determinant density using peptide-induced, Ld-restricted cytotoxic T lymphocytes. J. Exp. Med. 173, 849–858 (1991). 1901079 10.1084/jem.173.4.849 PMC2190800

[b25] Wherry E. J., Puorro K. A., Porgador A. & Eisenlohr L. C. The induction of virus-specific CTL as a function of increasing epitope expression: responses rise steadily until excessively high levels of epitope are attained. J. Immunol. 163, 3735–3745 (1999). 10490969

[b26] Song M. G. *et al.* *In vivo* imaging of differences in early donor cell proliferation in graft-versus-host disease hosts with different pre-conditioning doses. Mol. Cells 33, 79–86 (2012). 22228184 10.1007/s10059-012-2228-y PMC3887749

[b27] Jeon J. Y., Jung K. M., Chang J. & Choi E. Y. Characterization of CTL clones specific for single antigen, H60 minor histocompatibility antigen. Immune Netw. 11, 100–106 (2011). 21637387 10.4110/in.2011.11.2.100 PMC3100520

[b28] Intlekofer A. M. *et al.* Requirement for T-bet in the aberrant differentiation of unhelped memory CD8^+^ T cells. J. Exp. Med. 204, 2015–2021 (2007). 17698591 10.1084/jem.20070841 PMC2118697

[b29] Moon J. J. *et al.* Tracking epitope-specific T cells. Nat. Protoc. 4, 565–581 (2009). 19373228 10.1038/nprot.2009.9 PMC3517879

[b30] Wherry E. J. T cell exhaustion. Nat. Immunol. 12, 492–499 (2011). 21739672 10.1038/ni.2035

[b31] Shin H. *et al.* A role for the transcriptional repressor Blimp-1 in CD8^+^ T cell exhaustion during chronic viral infection. Immunity 31, 309–320 (2009). 19664943 10.1016/j.immuni.2009.06.019 PMC2747257

[b32] Wherry E. J. *et al.* Molecular signature of CD8^+^ T cell exhaustion during chronic viral infection. Immunity 27, 670–684 (2007). 17950003 10.1016/j.immuni.2007.09.006

[b33] Blackburn S. D., Shin H., Freeman G. J. & Wherry E. J. Selective expansion of a subset of exhausted CD8 T cells by alphaPD-L1 blockade. Proc. Natl Acad. Sci. USA 105, 15016–15021 (2008). 18809920 10.1073/pnas.0801497105 PMC2567485

[b34] Ha S. J. *et al.* Enhancing therapeutic vaccination by blocking PD-1-mediated inhibitory signals during chronic infection. J. Exp. Med. 205, 543–555 (2008). 18332181 10.1084/jem.20071949 PMC2275378

[b35] Kurachi M. *et al.* Chemokine receptor CXCR3 facilitates CD8^+^ T cell differentiation into short-lived effector cells leading to memory degeneration. J. Exp. Med. 208, 1605–1620 (2011). 21788406 10.1084/jem.20102101 PMC3149224

[b36] Ehst B. D., Ingulli E. & Jenkins M. K. Development of a novel transgenic mouse for the study of interactions between CD4 and CD8 T cells during graft rejection. Am. J. Transplant. 3, 1355–1362 (2003). 14525595 10.1046/j.1600-6135.2003.00246.x

[b37] Joshi N. S. *et al.* Inflammation directs memory precursor and short-lived effector CD8^+^ T cell fates via the graded expression of T-bet transcription factor. Immunity 27, 281–295 (2007). 17723218 10.1016/j.immuni.2007.07.010 PMC2034442

[b38] D'Souza W. N. & Hedrick S. M. Cutting edge: latecomer CD8 T cells are imprinted with a unique differentiation program. J. Immunol. 177, 777–781 (2006). 16818730 10.4049/jimmunol.177.2.777 PMC3137433

[b39] Marzo A. L. *et al.* Initial T cell frequency dictates memory CD8^+^ T cell lineage commitment. Nat. Immunol. 6, 793–799 (2005). 16025119 10.1038/ni1227 PMC2849311

[b40] Zehn D., Lee S. Y. & Bevan M. J. Complete but curtailed T-cell response to very low-affinity antigen. Nature 458, 211–214 (2009). 19182777 10.1038/nature07657 PMC2735344

[b41] Wherry E. J., Blattman J. N., Murali-Krishna K., van der Most R. & Ahmed R. Viral persistence alters CD8 T-cell immunodominance and tissue distribution and results in distinct stages of functional impairment. J. Virol. 77, 4911–4927 (2003). 12663797 10.1128/JVI.77.8.4911-4927.2003 PMC152117

[b42] Badovinac V. P. & Harty J. T. Manipulating the rate of memory CD8^+^ T cell generation after acute infection. J. Immunol. 179, 53–63 (2007). 17579021 10.4049/jimmunol.179.1.53

[b43] van Faassen H. *et al.* Reducing the stimulation of CD8^+^ T cells during infection with intracellular bacteria promotes differentiation primarily into a central (CD62L^high^CD44^high^) subset. J. Immunol. 174, 5341–5350 (2005). 15843531 10.4049/jimmunol.174.9.5341

[b44] Vezys V. *et al.* Continuous recruitment of naive T cells contributes to heterogeneity of antiviral CD8 T cells during persistent infection. J. Exp. Med. 203, 2263–2269 (2006). 16966427 10.1084/jem.20060995 PMC2118117

[b45] Obar J. J. *et al.* CD4^+^ T cell regulation of CD25 expression controls development of short-lived effector CD8^+^ T cells in primary and secondary responses. Proc. Natl Acad. Sci. USA 107, 193–198 (2010). 19966302 10.1073/pnas.0909945107 PMC2806751

[b46] Khanolkar A., Fuller M. J. & Zajac A. J. CD4 T cell-dependent CD8 T cell maturation. J. Immunol. 172, 2834–2844 (2004). 14978084 10.4049/jimmunol.172.5.2834

[b47] Nakanishi Y., Lu B., Gerard C. & Iwasaki A. CD8^+^ T lymphocyte mobilization to virus-infected tissue requires CD4^+^ T-cell help. Nature 462, 510–513 (2009). 19898495 10.1038/nature08511 PMC2789415

[b48] Kouskoff V., Signorelli K., Benoist C. & Mathis D. Cassette vectors directing expression of T cell receptor genes in transgenic mice. J. Immunol. Methods 180, 273–280 (1995). 7714342 10.1016/0022-1759(95)00002-r

