# Supplementary material for: Memory programming in CD8+ T-cell differentiation is intrinsic and is not determined by CD4 help
Source: Nat Commun. 2015 Aug 14;6:7994. doi: 10.1038/ncomms8994 (PMC4557278; doi:10.1038/ncomms8994)
Supplement: Supplementary Information — Supplementary Figures 1-9 [file ncomms8994-s1.pdf]

Supplementary Figure 1

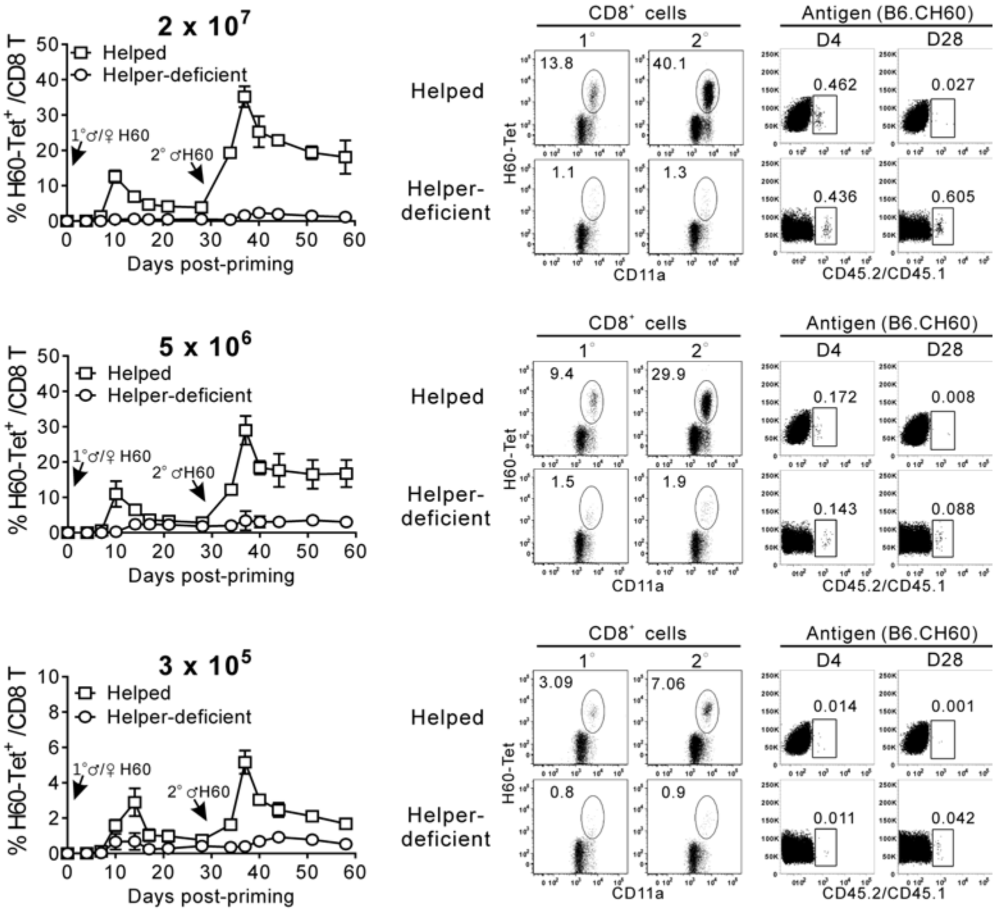

Supplementary Figure 1. Memory expansion of helper-deficient CD8$^+$ T cells primed and boosted with various antigen doses. Longitudinal PBL-analyses of female B6 mice primed and boosted with various numbers (2×10^7, 5×10^6, and 3×10^5) of female or male B6.CH60. Female CD45.2$^+$ B6 mice were i. p. injected with different numbers of female CD45.1$^+$ B6.CH60 spleen cells for helper-deficient priming and were boosted with same numbers of male CD45.1$^+$ B6.CH60 spleen cells 30 days later. Female CD45.1$^+$ B6 mice were primed with and boosted with same doses of male CD45.2$^+$ B6.CH60 spleen cells in parallel as helped control groups. PBLs from the immunized mice were analyzed periodically for detection of H60-tetramer-binding CD8 T cells and frequencies of H60-tetramer-binding cells in blood CD8$^+$ T cells were plotted. Representative flow cytometric data obtained at 1°

peak (day 10 - 14 post-priming) and 2° peak (day 7 - 14 post-boosting) days are presented after gating for $CD8^+$ cells. Flow cytometric analyses were also performed to detect injected $CD45.1^+$ or $CD45.2^+$ primed cells in blood of helper-deficient or helped hosts, respectively, on days 4 and 28 post-priming. Representative flow cytometric data are presented with proportions of $CD45.1^+$ or $CD45.2^+$ cells in the PBL of the corresponding hosts denoted. Data shown are representative of two independent experiments (*n* =3/group/experiment).

Supplementary Figure 2

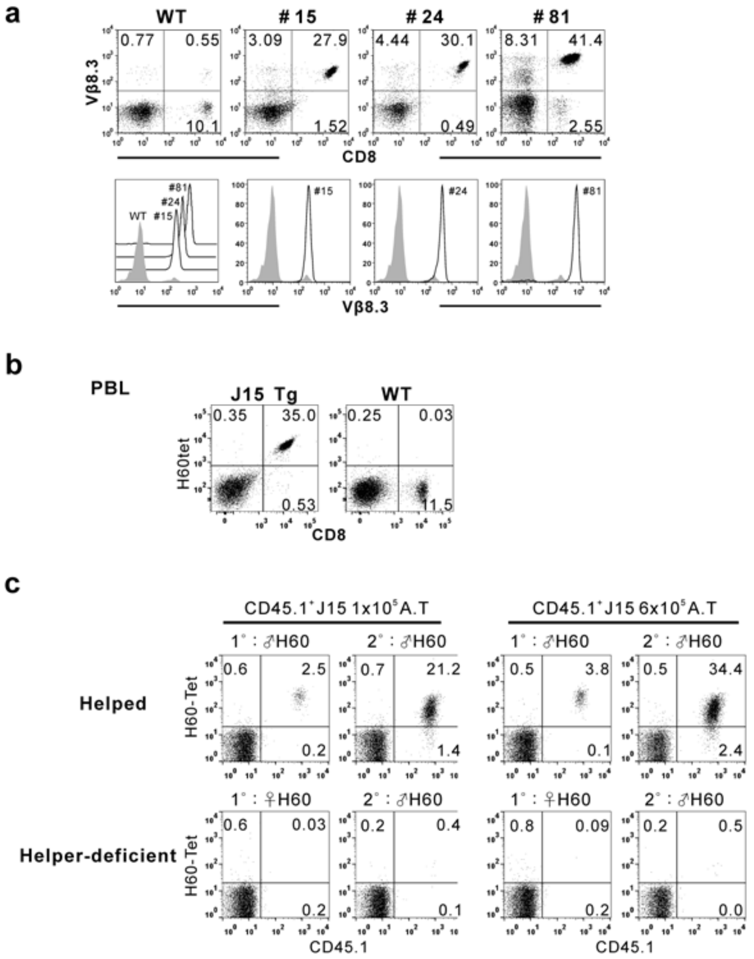

Supplementary Figure 2. Generation of transgenic TCR CD8$^+$ T cells (J15) specific for H60 and their helper-dependent responsiveness to H60-stimulation in adoptive hosts. (a) Flow cytometric analysis of PBLs from three different founder lines of TCR transgenic mice expressing TCRs specific for H60. Levels of Vβ8.3 expressed on PBLs of different TCR Tg founder lines (#15, #24, and #81; solid lines) and the wild type control B6 mice (gray-filled line) were compared after staining with anti-Vβ8.3 and anti-CD8 antibodies and following flow cytometric analysis. Line #15 was selected, based on comparability of surface Vβ8.3 levels with those expressed by wild type CD8$^+$ T cells, and was named J15. (b) Flow cytometric analysis of PBLs from J15 Tg or wild-type mice after staining for H60-tetramer

and anti-CD8. (c) $CD8^+$ T cells (1×10^5 or 6×10^5) purified from $CD45.1^+$ J15 mice were adoptively transferred to female B6 mice, primed under helper-deficient conditions and boosted 40 days later. Splenocytes from helper-deficient or helped hosts were harvested on day 10 post-priming (1°); those from helper-deficient-and-boosted, or helped-and-boosted hosts were harvested on day 7 post-boosting (2°). Splenocytes were analyzed by flow cytometry to detect H60-tetramer-binding cells in the spleen ($n = 2$). Data from at least three independent experiments are shown **(b, c).**

Supplementary Figure 3

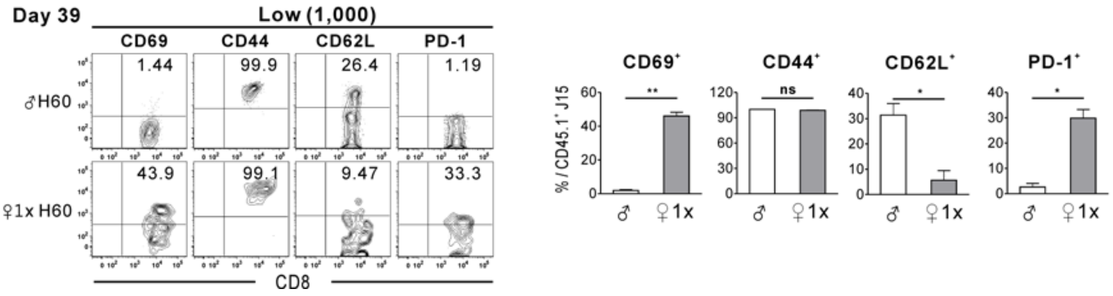

Supplementary Figure 3. Memory phenotypes of H60-specific CD8$^+$ T cells under different priming conditions in hosts adoptively transferred with low cell numbers. Phenotypic analysis on day 39 post-priming of splenic CD45.1$^+$ J15 CD8$^+$ T cells in the helped and helper-deficient hosts after low-number transfer (1×10^3). Mice in each experimental group were analyzed individually (*n* = 2/group). Flow cytometric results were analyzed after gating for CD45.1$^+$CD8$^+$ cells. Data representative of two independent experiments are shown. The values from all experiments were incorporated into plots. Data are presented as mean ± SEM. * $P < 0.05$, ** $P < 0.01$ determined by Student’s *t*-test.

Supplementary Figure 4

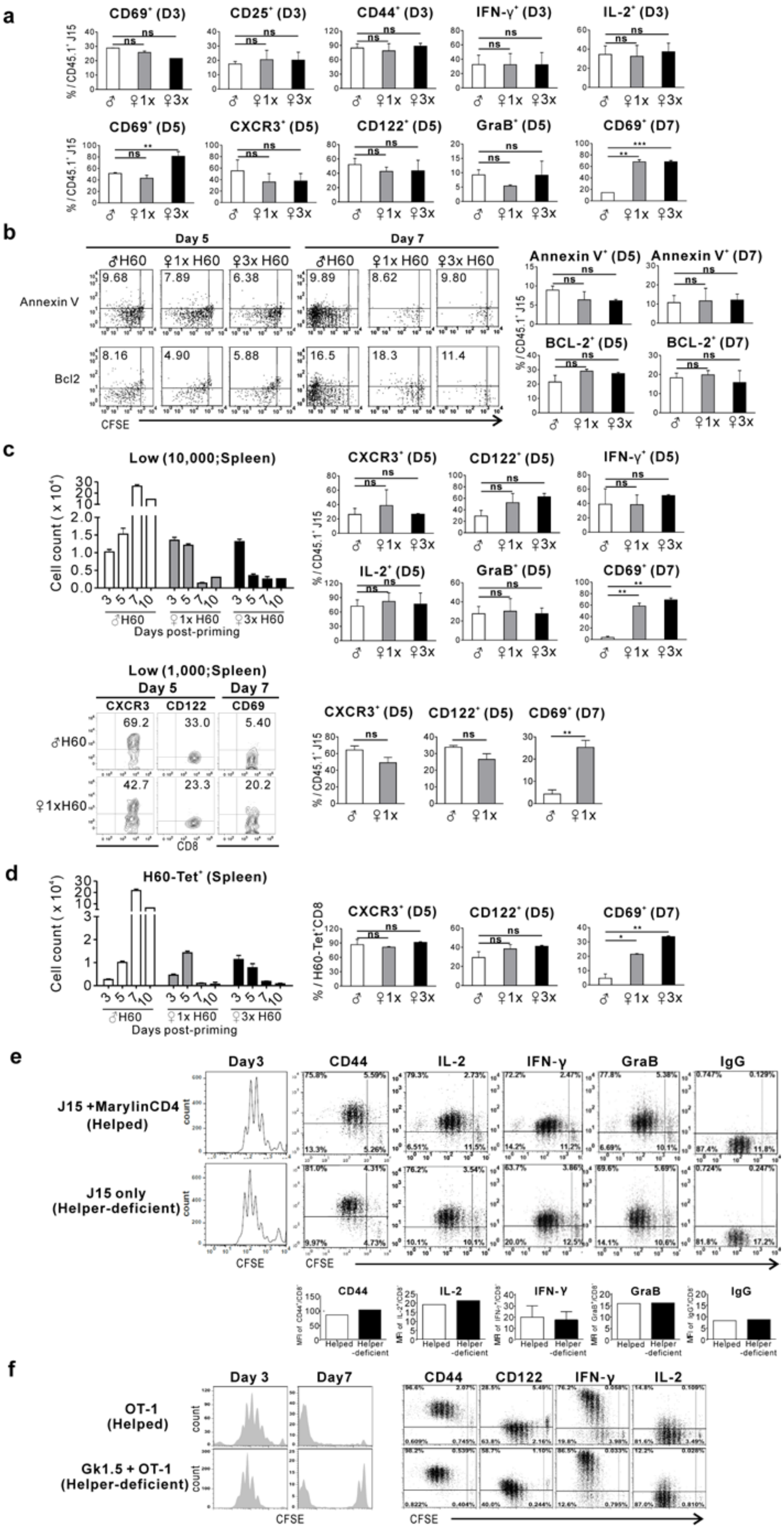

Supplementary Figure 4. Effector differentiation of helper-deficient CD8$^+$ T cells specific for H60 or Ova. **(a)** Percentages of cells positive for activation markers and effector molecules in J15 CD8$^+$ T cells present in the adoptive hosts transferred in high numbers. **(b)** Annexin-V-staining and Bcl-2-expression were compared between helper-deficient and helped J15 CD8$^+$ T cells. **(c, d)** Dynamics and phenotypes of **(c)** J15 CD8$^+$ T cells present in the spleens of adoptive hosts transferred in low numbers (10,000 and 1,000) and **(d)** polyclonal H60-tetramer binding CD8$^+$ T cells purified from spleens of primed B6 mice. **(e)** J15 CD8$^+$ T cells labeled with CFSE were incubated with peptide-pulsed DCs (H60 + HY-Dby) in the presence (helped) or absence (helper-deficient) of Marylin CD4$^+$ T cells for 3 days. The CFSE and phenotypic profiles of J15 CD8$^+$ cells were then analyzed. Mean fluorescence intensity (MFI) of each group is plotted. **(f)** Female B6 CD45.1$^+$ congenic mice were treated twice with GK1.5 or control Ig at 2-day intervals before adoptive transfer with CFSE-labeled OT-1 CD8$^+$ T cells (1×10^6). Hosts were then primed with spleen cells from Ova-Tg mice (2×10^7) 1 day after the adoptive transfer. Splenocytes were harvested from the primed hosts on days 3 and 7 for flow cytometric analysis. CFSE-profiles and flow cytometric data were obtained after gating CD45.1$^-$ CD8$^+$ cells. Data are representative of two **(c–f)** or three **(a, b)** independent experiments (*n* = 2/group/experiment). Data are presented as means ± SEM. * $P < 0.05$, ** $P < 0.01$, *** $P < 0.001$ determined by Student's *t*-test.

Supplementary Figure 5

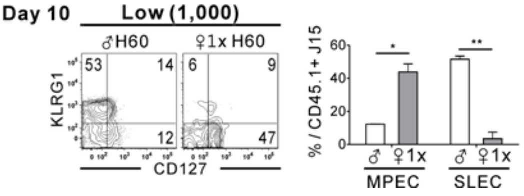

Supplementary Figure 5. SLEC and MPEC Differentiation of J15 CD8$^+$ T cells in the adoptive hosts transferred with low cell numbers. One thousand CD45.1$^+$J15 CD8$^+$ T cells were adoptively transferred into female B6 mice before priming with male or female B6.CH60 splenocytes. J15 CD8 T cells were analyzed on day 10 post-priming with gating on CD45.1$^+$ cells. Representative flow cytometry data, and plots of the proportions of short-lived effector cell (SLEC) and memory precursor effector cell (MPEC) populations in J15 CD8$^+$ T cells are shown. Data are representative of two independent experiments (n = 2/group/experiment) and are expressed as means ± SEM. * $P < 0.05$, ** $P < 0.01$, *** $P < 0.001$ determined by Student's t-test.

Supplementary Figure 6

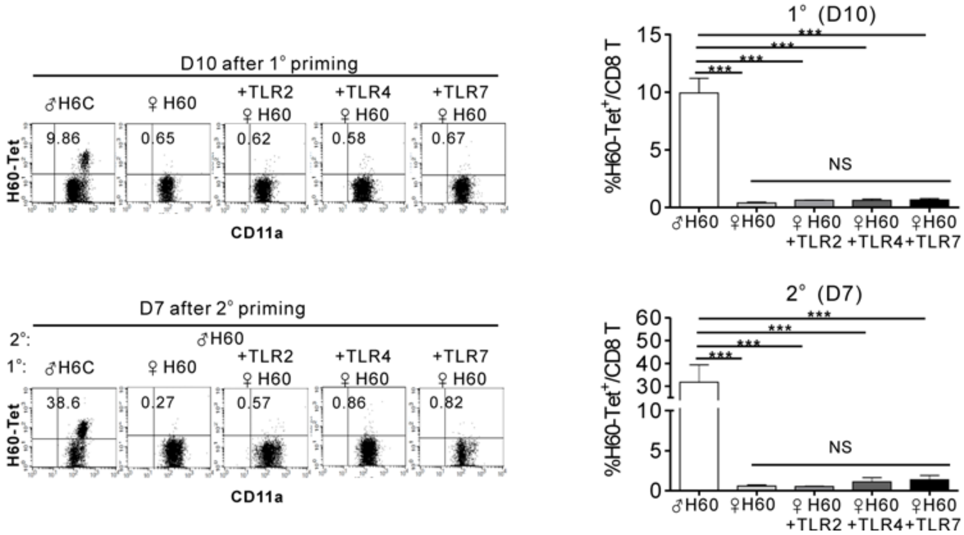

Supplementary Figure 6. Inflammation induced by TLR ligand-supplementation did not lead to memory cell generation by helper-deficient CD8$^+$ T cells. Helper-deficient B6 female mice were i.p. injected with agonists of TLR2 (Pam3CSK4, 50 μg; Invivogen, San Diego, CA. USA), TLR4 (LPS, 100 μg; Sigma-Aldrich, St. Louis, MO. USA), or TLR7 (loxoribine, 100 μg; Invivogen) on day 1 post-priming and boosted with male B6.CH60 splenocytes 30 days later. Control mice primed with either male (helped) or female B6.CH60 splenocytes (helper-deficient) without the TLR ligand-supplementation (PBS-treated) were boosted in parallel. PBLs from the immunized and boosted mice were longitudinally analyzed by flow cytometry after staining with H60-tetramer, and anti-CD11a and anti-CD8 mAbs. Representative flow cytometric data on day 10 post-priming and day 7 post-boosting are shown. Corresponding frequencies of H60-tetramer-positive cells in blood CD8$^+$ T cells are plotted. Data are representative of three independent experiments (n = 3/group/experiment) and are presented as means ± SEM. * P <0.05, ** P <0.01, *** P < 0.001 determined by Student's t-test.

Supplementary Figure 7

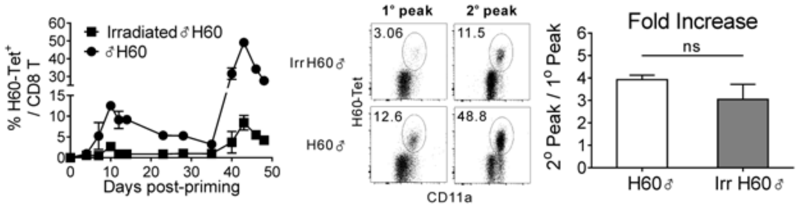

Supplementary Figure 7. Priming with irradiated splenocytes did not enhance memory cell generation of CD8$^+$ T cells under helped condition. Helped priming was performed by i.p. injection of lethally irradiated (2,000 cGy) or live male B6.CH60 splenocytes. The helped mice were boosted with live male cells as in the original protocol on day 35 post-priming. PBLs from the immunized and boosted mice were longitudinally analyzed by flow cytometry after staining with H60-tetramer, and anti-CD11a and anti-CD8 mAbs. Plots of frequencies of H60-tetramer-binding cells in CD8$^+$ T cells and representative flow cytometric data (day 10 post-priming and day 7 post-boosting) are shown. Fold increases in secondary peak compared to primary peak frequencies were plotted. Data represent two independent experiments (n = 3/group/experiment) and are presented as means ± SEM. P <0.05 was considered to indicate statistical significance.

Supplementary Figure 8

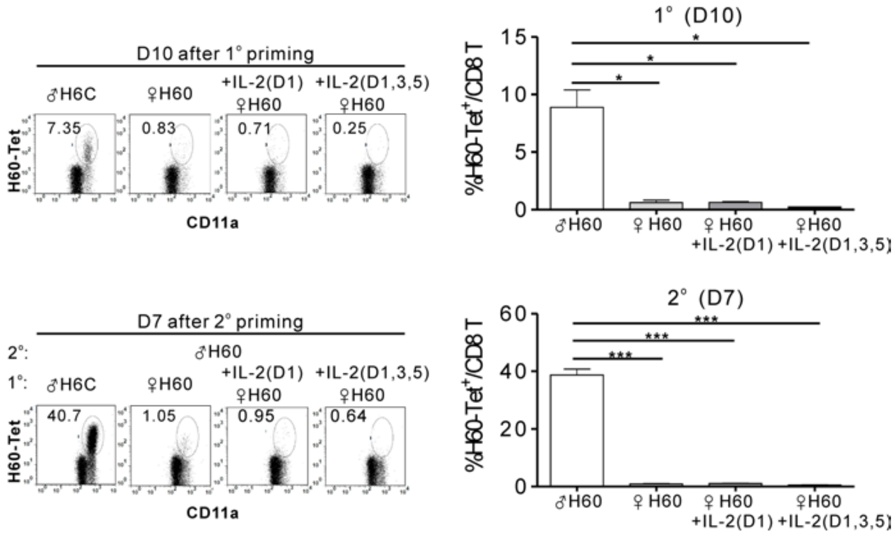

Supplementary Figure 8. Supplementation with IL-2 did not lead to memory cell generation by helper-deficient CD8$^+$ T cells. Helper-deficient B6 female mice were i.p. injected with recombinant mouse IL-2 (5 μg; Peprotech, RockyHill, NJ, USA) on day 1 (D1), or days 1, 3, and 5 (D1, 3, 5) after priming with female B6.CH60 splenocytes. Animals were then boosted with male B6.CH60 splenocytes 30 days later. Control mice were primed with either male (helped) or female (helper-deficient) B6.CH60 splenocytes without IL-2-supplementation and boosted in parallel. PBLs from the immunized and boosted mice were longitudinally analyzed by flow cytometry after staining with H60-tetramers, and anti-CD11a and anti-CD8 mAbs. Representative flow cytometric data obtained on day 10 post-priming and day 7 post-boosting, and plots of corresponding frequencies of H60-tetramer-positive cells in CD8$^+$ T cells are shown. Data are representative of two independent experiments (n = 3/group/experiment) and presented as means ± SEM. * P <0.05, ** P <0.01, *** P <0.001 determined by Student's t-test.

Supplementary Figure 9

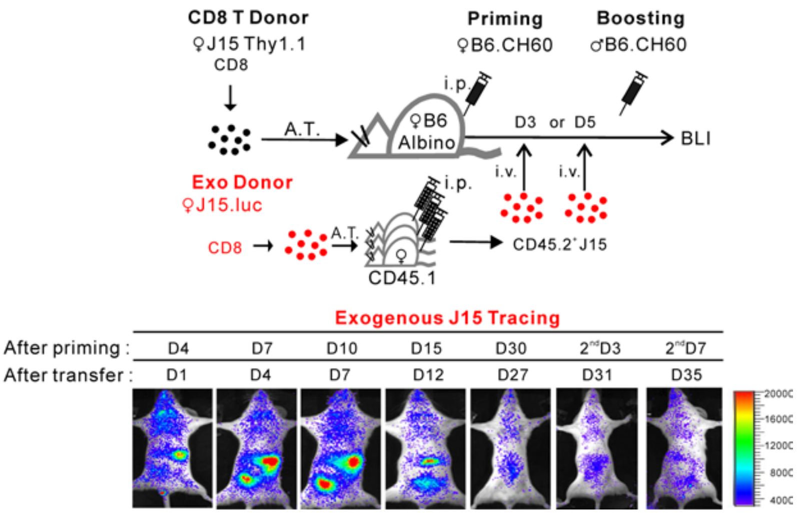

Supplementary Figure 9. Distribution of supplemented exogenous helper-deficient J15 CD8$^+$ T cells in helper-deficient primed hosts. Two groups of adoptive hosts (B6-albino hosts with Thy1.1$^+$ J15 CD8$^+$ T cells, and CD45.1$^+$ hosts with J15-LucTg CD8$^+$ T cells) were primed on the same day under helper-deficient conditions. J15-LucTg CD8$^+$ T cells (CD45.2$^+$) purified from three helper-deficient hosts on day 3 post-priming were injected i.v. to helper-deficient B6-albino hosts. Localization of J15-LucTg CD8$^+$ T cells was monitored longitudinally in the helper-deficient albino hosts beginning at 1 day after injection i.v. for 35 days. Images are representative of four mice from two independent experiments (*n*= 2/experiment).
